# Danthron as a novel IL-6R agonist promotes thrombopoiesis via the SRC/RAS/MAPK pathway

**DOI:** 10.3389/fimmu.2026.1730028

**Published:** 2026-03-27

**Authors:** Rui Liao, Jiesi Luo, Xiaoxuan Li, Xiaoqin Tang, Miao Huang, Ling Zhou, Long Yun, Zhixuan Liu, Peilian Jiang, Xinle Wang, Xiaolin Gan, Long Wang, Jianming Wu

**Affiliations:** 1 Sichuan Key Medical Laboratory of New Drug Discovery and Druggability, Luzhou Key Laboratory of Activity Screening and Druggability Evaluation for Chinese Materia Medica, School of Pharmacy, Southwest Medical University, Luzhou, China; 2 School of Basic Medical Sciences, Southwest Medical University, Luzhou, Sichuan, China; 3 Department of Pharmacy, The Second People’s Hospital of Yibin, Yibin, China; 4 School of Pharmacy, Chengdu University of Traditional Chinese Medicine, Chengdu, China; 5 Key Laboratory of Medical Electrophysiology, Ministry of Education and Medical Electrophysiological Key Laboratory of Sichuan Province, (Collaborative Innovation Center for Prevention of Cardiovascular Diseases), Institute of Cardiovascular Research, Southwest Medical University, Luzhou, China

**Keywords:** danthron, IL-6R, megakaryocyte, platelet, SRC/RAS/MAPK, thrombocytopenia

## Abstract

**Background:**

This study aimed to investigate the effects of danthron on promoting megakaryocyte (MK) differentiation and alleviating thrombocytopenia, as well as to elucidate its underlying mechanisms.

**Methods:**

Cell proliferation and apoptosis of Meg-01 and K562 cells were evaluated by CCK-8, LDH release, and apoptosis assays. MK differentiation was assessed by Giemsa staining, phalloidin staining, and flow cytometry. A thrombocytopenia mouse model was induced by 4 Gy irradiation and treated intraperitoneally with danthron for 12 days, followed by hematological, histopathological, MK differentiation, and tail bleeding analyses. Potential targets and pathways were explored using network pharmacology, molecular docking, GO/KEGG enrichment, Western blotting, and inhibitor validation.

**Results:**

Danthron effectively promoted MK differentiation *in vitro*, inducing the expression of MK differentiation–related transcription factors NF-E2, RUNX1, MEIS1, and HIF-1β, without exhibiting significant cytotoxicity. In addition, danthron markedly accelerated the recovery of MK progenitors, MKs, and platelet levels in thrombocytopenic mice, and shortened tail bleeding time. These effects were associated with danthron directly targeting IL-6R and activating the downstream SRC/RAS/MAPK signaling pathway, whereas inhibition of IL-6R or ERK abrogated these effects.

**Conclusion:**

This study uncovered danthron as a novel natural small-molecule agonist of IL-6R and demonstrated its therapeutic potential in thrombocytopenia. The effect of danthron is mediated by its capacity to promote MK differentiation and induce platelet production through targeting of IL-6R and activation of the downstream SRC/RAS/MAPK signaling pathway. These results highlight danthron as a promising candidate for thrombocytopenia therapy and underscore the therapeutic potential of targeting IL-6R signaling for hematopoietic regulation.

## 1 Introduction

Thrombocytopenia is a prevalent complication among cancer patients undergoing radiotherapy and chemotherapy. It is accompanied by a decrease in platelet count and an increased risk of bleeding, significantly affecting treatment efficacy and patient outcomes (1). Radiation-induced thrombocytopenia (RIT) represents a critical hematopoietic concern in acute radiation syndromes, frequently leading to increased morbidity and mortality among affected patients (2, 3). Current therapeutic options for thrombocytopenia, including glucocorticoids, thrombopoietin (TPO) receptor agonists (TPO-RAs), and immunoglobulins, demonstrate limited efficacy and are associated with various adverse effects. For instance, glucocorticoids may contribute to the development of diabetes mellitus and osteoporosis (4, 5), whereas TPO-RAs, such as eltrombopag and romiplostim, can lead to complications including myelofibrosis, elevated transaminases, and thromboembolism (6). Additionally, the availability of immunoglobulins is often limited, and their use can result in headaches and allergic reactions (6). Consequently, the management of thrombocytopenia remains a significant clinical challenge, underscoring the urgent need for safer and more effective therapeutic alternatives.

Megakaryocyte (MK) production involves the proliferation and differentiation of hematopoietic stem cells (HSCs), ultimately culminating in platelet generation (7). This process is predominantly regulated by TPO, a key modulator of platelet homeostasis (8), which activates various signaling pathways, including RAS/MAPK and JAKs/STATs, upon binding to its receptor, c-Mpl (9). However, studies involving TPO^-^/^-^ or c-Mpl^-^/^-^ mice indicate that TPO alone does not exclusively govern thrombopoiesis (10–12). Therefore, there is an urgent need to identify a novel, highly effective, and low-toxicity receptor agonist for the treatment of thrombocytopenia. The interleukin-6/interleukin-6 receptor (IL-6/IL-6R) pathway not only plays a pivotal role in MK maturation and the regulation of blood platelet counts (13), but also synergizes with TPO to promote the proliferation of TPO-dependent MK colony-forming units (CFU-Meg) (14). These findings suggest that modulation of the IL-6/IL-6R pathway may confer therapeutic benefits for patients suffering from thrombocytopenia.

Rhubarb is a classical herbal medicine in traditional Chinese medicine (TCM) and ethnomedicine. As early as in the classical Chinese text *Jin Kui Yao Lue*, rhubarb was documented for its hemostatic properties, being prescribed to rapidly control bleeding and restore platelet levels. For centuries, rhubarb has also been widely prescribed for centuries to “activate blood circulation,” “remove blood stasis,” and “cool the blood to stop bleeding” (15, 16). These traditional indications are closely associated with blood-related disorders, including hematological imbalance and bleeding syndromes (17). In the theory of TCM, “blood stasis” and “blood deficiency” are pathophysiological conditions that overlap with impaired hematopoiesis, coagulation abnormalities, and thrombocytopenia in modern medicine (18, 19). Thus, rhubarb has long been valued as a blood-regulating herb in ethnopharmacological practice. Phytochemical investigations have identified anthraquinones, such as danthron, emodin, and rhein, as the major active constituents of rhubarb (20). These compounds have various pharmacological properties, including anti-inflammatory, anti-atherosclerotic, metabolic regulation and therapeutic activities for intracerebral hemorrhage (21, 22). However, despite rhubarb’s traditional relevance to hematological disorders, studies addressing the role of its anthraquinone derivatives in hematopoietic regulation remain limited. In particular, the effects of anthraquinones on MK differentiation and platelet production have not been systematically elucidated.

To address this gap, we investigated the hematopoietic activity of danthron, a representative anthraquinone from rhubarb. We identified danthron as a novel small-molecule agonist of IL-6R for the first time and elucidated its role in promoting MK differentiation and platelet production. By integrating *in vitro* assays, an *in vivo* radiation-induced thrombocytopenia model, and molecular mechanism studies, we demonstrate that danthron activates the IL-6R/SRC/RAS/MAPK pathway to restore platelet counts. This study not only provides pharmacological evidence supporting the ethnomedicinal use of rhubarb in hematological disorders but also proposes danthron as a promising lead compound for developing novel IL-6R-targeted therapeutics for thrombocytopenia.

## 2 Materials and methods

### 2.1 Chemicals

Danthron (1,8-dihydroxyanthraquinone, DST210708-018) was obtained from Chengdu Desite Biotechnology Co., Ltd. (Chengdu, China). The compound was verified by HPLC with a purity of 99.3%. For experimental use, danthron was dissolved in DMSO to prepare stock solutions and stored at −20 °C until further use. PMA was obtained from Macklin (Shanghai, China).

### 2.2 Cell culture

Meg-01 and K562 cells were obtained from the American Type Culture Collection (Bethesda, MD, United States), which were cultured in RPMI-1640 complete medium (Gibco, Thermo Fisher Scientific, Waltham, MA, United States) supplemented with 10% fetal bovine serum (FBS, CAT: SP002030100, Sperikon Biotechnology Co., LTD, Sichuan, China) and 1% (v:v) Penicillin-Streptomycin Solution (Sperikon Biotechnology Co., LTD,Sichuan, China). Cells were maintained in a humidified incubator at 37 °C with 5% CO_2_.

### 2.3 Cell proliferation assay

The proliferation of Meg-01 and K562 cells was assessed using a Cell Counting Kit-8 (CCK-8) assay kit (Dojindo, Kumamoto, Japan). Cells were seeded in 96-well plates at a density of 4×10^3^ per well and treated with different concentrations of danthron (2, 4 and 8 μM) for 1, 3 and 5 days. When the intervention was completed, 20 μL of CCK-8 assay reagent was added to each well and incubated at 37 °C for 2 hours. Subsequently, absorbance was measured using an Epoch Multi-Volume Spectrophotometer System (BioTek, Winooski, VT, USA) at a wavelength of 450 nm. Each experimental group included more than 3 wells. The absorbance values were normalized to those of the corresponding control group at each time point. First, the mean absorbance value of the control group at each time point (Day 1, 3, and 5) was calculated. Then, the absorbance values of each danthron-treated well at each time point were divided by the mean absorbance value of the corresponding time-matched control group. The resulting ratio was multiplied by 100 to be presented as “Cell proliferation (% of time-matched control)”.

### 2.4 Lactate dehydrogenase assay

Lactate dehydrogenase (LDH) release was measured using a LDH assay kit (CAT: C0017, Beyotime, Shanghai, China). Meg-01 and K562 cells were cultured at the same density in 96-well plates and treated with different concentrations of danthron. LDH release was measured on days 1, 3, and 5 after danthron treatment. The maximum LDH release control group was also established. At the specified time points, cells in the maximum LDH release control group were first treated with lysis solution cells at 37 °C for 1 hour to ensure complete LDH release. Subsequently, a series of LDH detection reagents were added to each well and allowed to react for 30 minutes. Finally, absorbance was measured at 490 nm.

### 2.5 Cell apoptosis assay

Cell apoptosis was assessed using an Annexin V-FITC apoptosis detection kit (CAT: C1062M, Beyotime, Shanghai, China). Meg-01 and K562 cells were cultured in 6-well plates at a density of 1×10^4^ cells per well with and without the intervention of danthron (2, 4 and 8 μM). After five days of treatment, cells were collected and centrifuged at 1500 rpm for 5 minutes, then washed once with PBS and centrifuged again at the same speed for 5 minutes. The supernatant was removed and 195 μL Annexin V-FITC binding buffer was added to gently suspend the cells. Next, 5 μL of FITC-conjugated Annexin V and 10 μL of PE-conjugated PI staining solution were added to each group and mixed thoroughly. The cells were incubated in the dark at room temperature (20-25 °C) for 10–20 minutes. The apoptosis of cells was detected by BD FACSCanto II flow cytometer (BD Biosciences, San Jose, CA, USA). FlowJo was used to analyze the results.

### 2.6 Cell morphology observation

K562 and Meg-01 cells were cultured at the same density in cells plates with intervention of danthron (2, 4, and 8 μM) and PMA (1.25 nM) for 5 days. Using a microscope (NIKON, Japan), at least three random fields were selected to observe the different morphologies of cells at 20× magnification.

### 2.7 Giemsa staining

After 5 days of drug intervention, K562 and Meg-01 cells were collected, centrifuged, and washed once with PBS before being centrifuged again. The cells were then swollen with KCl for about 30 seconds, fixed in a mixture of methanol and acetic acid (3:1) for 5 minutes, and centrifuged at 1500 rpm for 3 minutes. A 30 μL aliquot was drawn onto a pre-washed glass slide. Giemsa Stain Solution (concentrate: diluent = 1:9, Solarbio, CAT: G1010) was added and allowed to stain for 8 minutes. After that, the glass slide was rinsed with distilled water for 30 seconds. After drying, nuclear morphology was observed using a microscope.

### 2.8 Phalloidin staining

Two types of cells were treated with danthron (2, 4 and 8 μM) and PMA (1.25 nM) for 5 days. The cell washing steps were performed as described for Giemsa staining. The washed cells were then fixed with 4% paraformaldehyde (Biosharp, China) for 10 minutes. After that, the cell suspension was centrifuged at 600 g for 3 min, and the cells were fixed on a glass plate washed with pure water in advance. The cells were washed 2–3 times with PBS and then permeabilized with a 0.5% Triton X-100 solution for 5 minutes. The cytoskeleton was stained with 0.5% TRITC phalloidin working solution (Solarbio, Beijing, China) in the dark for 1 hour. Finally, the nucleus was stained with 100 nM DAPI (Solarbio, Beijing, China) for 30 seconds. The images of each sample were observed using a fluorescence inverted microscope (Leica, Wetzlar, Germany).

### 2.9 Analysis of CD41 and CD42b expression

K562 and Meg-01 cells were cultured at the same density in cell culture plates and treated with danthron (2, 4, and 8 μM), PMA (1.25 nM) and SCH772984 (ERK inhibitor) (Topscience, Shanghai, China) or Tocilizumab (IL-6R inhibitor) (MedChemExpress, USA) for 5 days. Following the cleaning protocol outlined previously, the cells were incubated with FITC-conjugated anti-CD41 and PE-conjugated anti-CD42b (4A Biotech Co., Lta, China) in the dark at room temperature for 30 minutes. Staining was then halted by adding 300 μL of cold PBS solution. The ratio of CD41^+^/CD42b^+^ cells was assessed using a BD FACSCanto II flow cytometer (BD Biosciences, San Jose, CA, USA), and the results were analyzed with FlowJo software.

### 2.10 Cell ploidy analysis

Cells were collected and washed with cold PBS, then fixed overnight at 4 °C with 70% pre-cooled ethanol solution. Before the experiment, the cells were centrifuged to remove ethanol and washed once with cold PBS. After the PBS was removed, PI stain solution (BD Biosciences, CAT:550825, USA) was used for low temperature staining in the dark for 20 minutes. The ploidy of each group of cells was assessed using a BD FACSCanto II flow cytometer, and the results were analyzed with FlowJo software.

### 2.11 Animals

Specific pathogen-free Kunming (KM) mice (8–10 weeks old), male and female mice with equal body weight distribution (20.0 ± 2) g, were acquired from Dashuo Biotechnology Co., Ltd. (Chengdu, China). The experimental mice were housed on a standard diet and subjected to a 12-hour light/dark cycle. The temperature in the housing facility maintained between 20 and 26 °C, with humidity levels at 50 to 60%. All animal experiments were conducted in accordance with the regulations set forth by the Experimental Animal Ethics Committee of Southwest Medical University (License NO. 20220825-011). After 7 days of acclimatization feeding, the mice were assigned to the following groups: control, model (RIT), model + rhTPO (3000 U/kg, positive control) and model + danthron (2.5, 5 and 10mg/kg). Each group consisted of 4 female and 4 male mice, totaling 8 mice per group. All experimental groups, except the control group, received 4 Gy X-ray irradiation to induce thrombocytopenia in mice.

### 2.12 Peripheral blood routine analysis

After radiation modeling, mice in each group received daily intraperitoneal injections of physiological saline (0.1 mL/10 mg), rhTPO and danthron. Peripheral blood samples of 40 μL were collected from the posterior orbital venous plexus of mice on days 0 (baseline), 1, 3, 7, 10, and 12. These samples were mixed with 160 μL of anticoagulant solution. The hematological parameters were measured using a blood cell analyzer (Sysmex XT-1800i/2000IV; Kobe, Japan).

### 2.13 Assay of serum ALT, AST and CK levels in mice

24 hours after the final administration, blood samples were collected via retro-orbital bleeding and allowed to clot at room temperature. Serum was obtained by centrifugation at 3,000 × g for 10 minutes and stored at −80 °C until analysis. Serum levels of alanine aminotransferase (ALT), aspartate aminotransferase (AST), and creatine kinase (CK) were measured using a fully automated clinical chemistry analyzer (Beckman Coulter AU680 clinical chemistry analyzer, USA) in the Clinical Laboratory of the Affiliated Hospital of Southwest Medical University. All assays were performed according to the manufacturer’s instructions and quality control procedures.

### 2.14 Flow cytometry analysis of bone marrow and spleen cells

Each group received intraperitoneal injections consecutively for 12 days. According to the guidelines of the Animal Ethics Committee of Southwest Medical University, mice were anesthetized with inhaled isoflurane using a calibrated precision vaporizer connected to an oxygen source. Anesthesia was induced in a closed induction chamber with 3–5% isoflurane (v/v) at an oxygen flow rate of approximately 0.5–1.0 L/min, and then maintained at 1.0–2.0% (v/v) during the surgical procedure. The depth of anesthesia was monitored by assessing pedal reflex, respiratory rate, and mucosal coloration. Following anesthesia, the spleen and femur were excised, and the femur was flushed with 1 mL of sterile normal saline to isolate bone marrow cells. One-third of the spleens were processed into single-cell suspensions, which were filtered with 200 mesh nylon mesh and 1 mL normal saline to obtain spleen cells. Red blood cells from both the bone marrow and spleen cells were removed using red blood cell lysate at low temperature. Subsequently, the cells were washed twice with PBS and 1×10^6^ cells were resuspended in 100 μL PBS. The samples were then incubated in the dark at room temperature with FITC-conjugated anti-CD41, PE-conjugated anti-CD61, and PE-conjugated anti-c-Kit for 20 minutes. The percentages of CD41^+^, CD61^+^, and c-Kit^+^ cells were analyzed using flow cytometry, with FlowJo software being used for data analysis.

### 2.15 Peripheral blood platelet flow cytometry

From the orbital venous plexus of each mouse, 50 µL of blood was collected into an EP tube containing 1 mL of sodium citrate. After centrifugation and addition of 500 µL of red blood cell lysis buffer, the cells were washed once with PBS. Each sample was then adjusted to a cell density of 1 × 10^6^ cells. Subsequently, samples were incubated with PE-conjugated anti-CD61 and FITC-conjugated anti-CD41 for 20 minutes. Flow cytometric analysis was then performed to determine the percentage of CD41^+^/CD61^+^ cells.

### 2.16 Immunofluorescence

Meg-01 cells were treated with danthron for 5 days. On day 5, the cells were collected and washed twice with PBS. The samples were then fixed with 1 mL of 4% paraformaldehyde for 15 minutes. Following fixation, the samples were permeabilized with 0.2% Triton X-100 for 15 minutes and blocked with 5% BSA for 25 minutes. NF-E2 and RUNX1 antibodies were incubated overnight at 4 °C. The following day, the antibodies were removed, and the cells were washed three times with PBS. The cells were then incubated with Rhodamine-labeled rabbit anti-goat IgG (H+L) (ZSGB-BIO, ZF-0317) for 1.5 hours at room temperature and washed three times with PBS. DAPI staining was performed for 30 seconds. Finally, the samples were observed using a fluorescence inverted microscope (Leica, Wetzlar, Germany) with a random field of view.

### 2.17 Network pharmacology, molecular docking, GO and KEGG enrichment analysis

The potential targets of drug action were identified using the SwissTargetPrediction and GeneCards databases, while thrombocytopenia-related targets were sourced from the DisGeNET and GeneCards databases. Subsequently, Venn diagrams were generated to identify common targets between the drugs and thrombocytopenia. The STRING_v12.0 database was employed to construct the protein-protein interaction (PPI) network. Cytoscape_v3.9.0 software was utilized to perform network analysis and identify core targets. The results of the Gene Ontology (GO) and Kyoto Encyclopedia of Genes and Genomes (KEGG) enrichment analyses were presented as bar charts and bubble charts, respectively, using the OmicShare Tools website. The docking process of drug and protein structures was carried out using Sybyl-X 2.0 software. Finally, Pymol_v2.5.2 was employed to visualize the molecular docking results.

### 2.18 Western blot

Meg-01 cells were seeded in 12-well plates at a density of 2×10^4^ cells per well and treated with or without danthron (2, 4 and 8 μM) for 5 days. On the 5th day, the cells were collected and washed once with PBS. After removing the PBS, an appropriate volume of cold RIPA lysis buffer (CST Lot: 26, Boston, MA, USA), supplemented with a phosphatase inhibitor (CAT No: C0002, TargetMol, USA), was added according to the number of cells in each group to lyse cells. The samples were placed at −80 °C overnight. Protein concentrations were determined using the Quick Start Bradford 1× Dye Reagent (Bio-Rad, CAT: 5000205, USA), with absorbance measured at 595 nm. Following this, 1/5 loading buffer (Epizyme Biomedical Technology Co., Ltd, CAT: LT101S, Shanghai, China) was added to each protein sample, which were then heated in a metal bath for 10 minutes and subsequently cooled at −20 °C. The protein samples were subjected to SDS-PAGE, followed by transfer to PVDF membranes. The PVDF membranes were blocked with a protein-free rapid blocking buffer (Epizyme Biomedical Technology Co., Ltd, LOT: 03792800, Shanghai, China) for 20 minutes and washed three times with PBST (PBS with 2% Tween-20). Next, the primary antibody was incubated at 4 °C overnight, and the PVDF membranes were washed three times with PBST. Following this, the secondary antibody was incubated at room temperature for 1 hour. Protein band imaging was performed using an ECL chemiluminescence detection kit (Proteintech, LOT: 20032561, Wuhan, China). The gray values of each group of protein band were analyzed using ImageJ software, with each protein tested at least three times. The following antibodies were used in the study: anti-IL6 (Lot: 66146-1-Ig), anti-NF-E2 (Lot: 66436-1-Ig), anti-RUNX1 (Lot: 25315-1-AP), and anti-GAPDH (Lot: 60004-1-AP) from Proteintech (Wuhan, China); anti-IL-6R (Lot: TD6466S), anti-RAS (Lot: T56672S), and anti-p-ERK (Lot: TA1015) from Abmart (Shanghai, China); anti-MEK (Lot: D1A5), anti-p-MEK (Lot: Ser217/221), anti-HIF-1β (Lot: D28F3), and anti-rabbit IgG (Lot: 4414) from Cell Signaling Technology (Boston, MA, USA); anti-MEISI (Lot: DF8102948) from Affinity (Melbourne Australia); anti-mouse IgG (Lot: DP04DF2Z7424) from Elabscience (Wuhan, China).

### 2.19 Statistical analysis

All experiments were performed in triplicate or more. Data are presented as mean ± standard deviation (SD) and analyzed using GraphPad Prism 8.0 (San Diego, CA, USA). The normality of data distribution was assessed using the Shapiro-Wilk test. For comparisons between two groups, Student’s *t*-test or the Mann-Whitney test was used, as appropriate. For comparisons among multiple groups, either one-way or two-way analysis of variance (ANOVA) was performed, followed by Tukey’s *post hoc* test for multiple comparisons. Statistical significance was set at *P* < 0.05.

## 3 Results

### 3.1 Danthron promotes MK differentiation of Meg-01 and K562 cells *in vitro*

**Figure 1:**
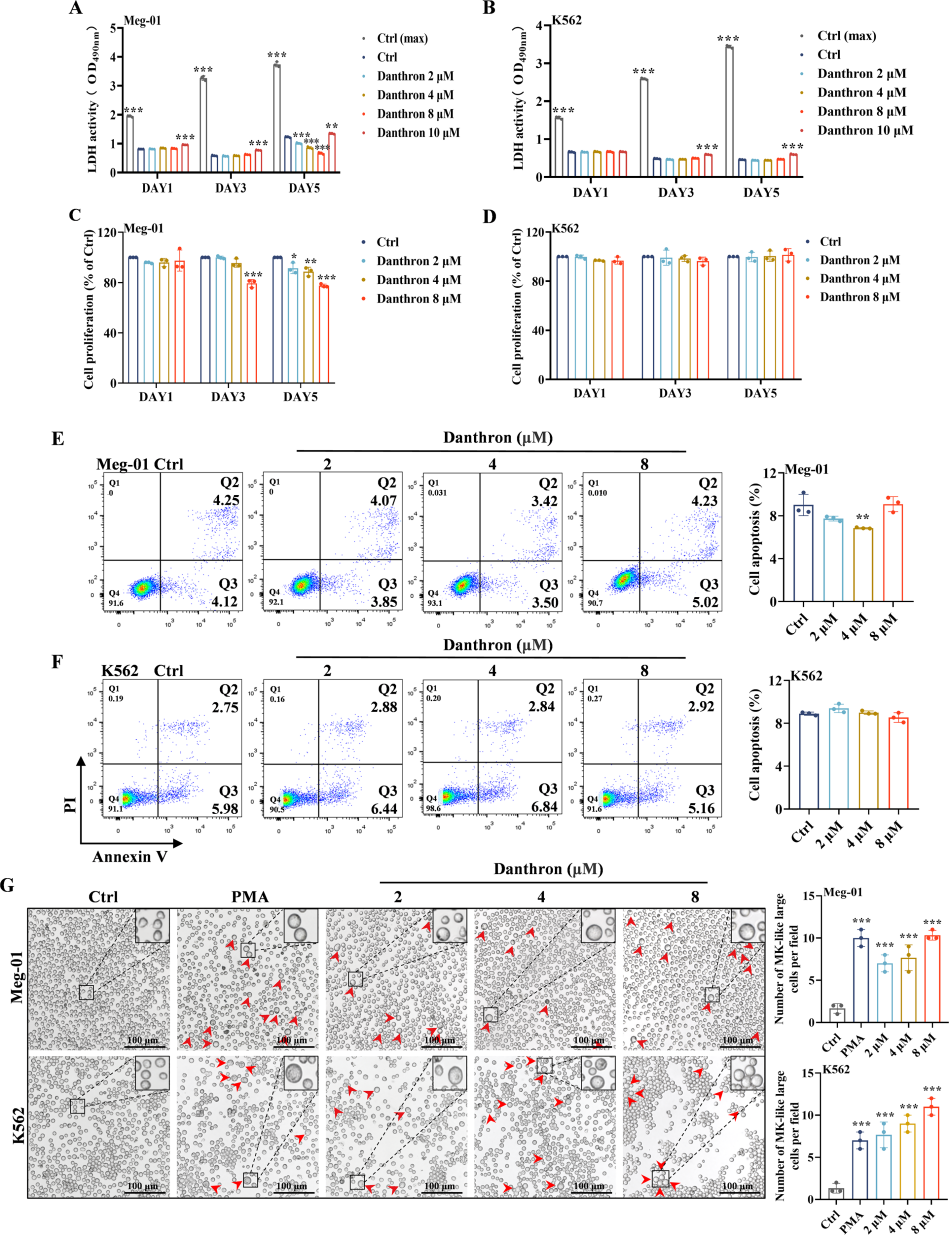
Danthron demonstrates no significant toxicity to Meg-01 and K562 cells *in vitro*. **(A-B)** LDH assays were conducted on days 1, 3, and 5 during danthron (2, 4, 8 and 10 μM) intervention in Meg01 and K562 cells. Ctrl (max) is the LDH maximum release control group. **(C-D)** The proliferation results of Meg-01 and K562 cells treated with danthron (2, 4 and 8 μM) for 1, 3 and 5 Days. The absorbance values were normalized to the corresponding control group at each time point (see Materials and Methods 2.3 for the specific normalization procedure). From day 1 to day 5, the number of viable cells in the Meg-01 and K562 control groups increased by 3.1-fold and 2.89-fold, respectively. **(E-F)** Cell apoptosis was analyzed by flow cytometry in Meg-01 and K562 cells treated with danthron (2, 4 and 8 μM). **(G)** The representative images of each group with different concentrations of danthron (2, 4 and 8 μM) on the 5th day. A preliminary quantitative assessment of megakaryocyte-like large cells based on morphological observations. PMA (1.25 nM) is positive control. Scar bar: 100 µm. All data are expressed as mean ± SD. n = 3 per group. *P < 0.05, **P < 0.01, and ***P < 0.001, vs. the Ctrl group.

We initially employed the well-established Meg-01 and K562 cell lines to investigate the capacity of danthron to promote MK differentiation and maturation *in vitro*, as these cell lines are widely recognized as standard models for studying MK differentiation (23, 24). Initially, we assessed the cytotoxicity and proliferative activity of Meg-01 and K562 cells treated with danthron over specified durations. The evaluation of the cytotoxic effect revealed no significant differences among the 2, 4, and 8 μM danthron-treated groups compared to the control group for K562 cells (**Figure 1B**). In the case of Meg-01 cells, toxicity did not differ among the 2, 4, and 8 μM danthron treatment groups on days 1 and 3. However, a notable inhibition of cellular LDH release was observed on day 5 (**Figure 1A**). Notably, treatment with 10 μM danthron exhibited significant toxicity in both cell lines (**Figures 1A, B**). Furthermore, there was no significant effect on the proliferation viability of K562 cells following treatment with 2, 4 and 8 μM danthron (**Figure 1D**). In contrast, the proliferation of Meg-01 cells was inhibited on day 5 of danthron treatment (**Figure 1C**), which may be attributed to enhanced differentiation of MK. However, additional analyses are required to establish a causal relationship between reduced proliferation and megakaryocytic differentiation. Additionally, no indications of apoptosis were observed in the danthron-treated group (**Figures 1E, F**). Based on the absence of overt cytotoxicity or apoptosis, concentrations of 2, 4, and 8 μM danthron were selected for subsequent *in vitro* experiments. To preliminarily ascertain whether 2, 4 and 8 μM danthron induced MK differentiation, we utilized phorbol 12-myristate 13-acetate (PMA, 1.25 nM), a compound previously reported to promote MK differentiation, as an *in vitro* positive control (25). After 5 days of treatment, a significant population of MK-like large cells was noted in the danthron-treated group, analogous to the PMA-treated group, while only a limited number were present in the control group (**Figure 1G**). Based on these findings, we selected the concentrations of 2, 4, and 8 μM danthron for further experimental investigations.

**Figure 2:**
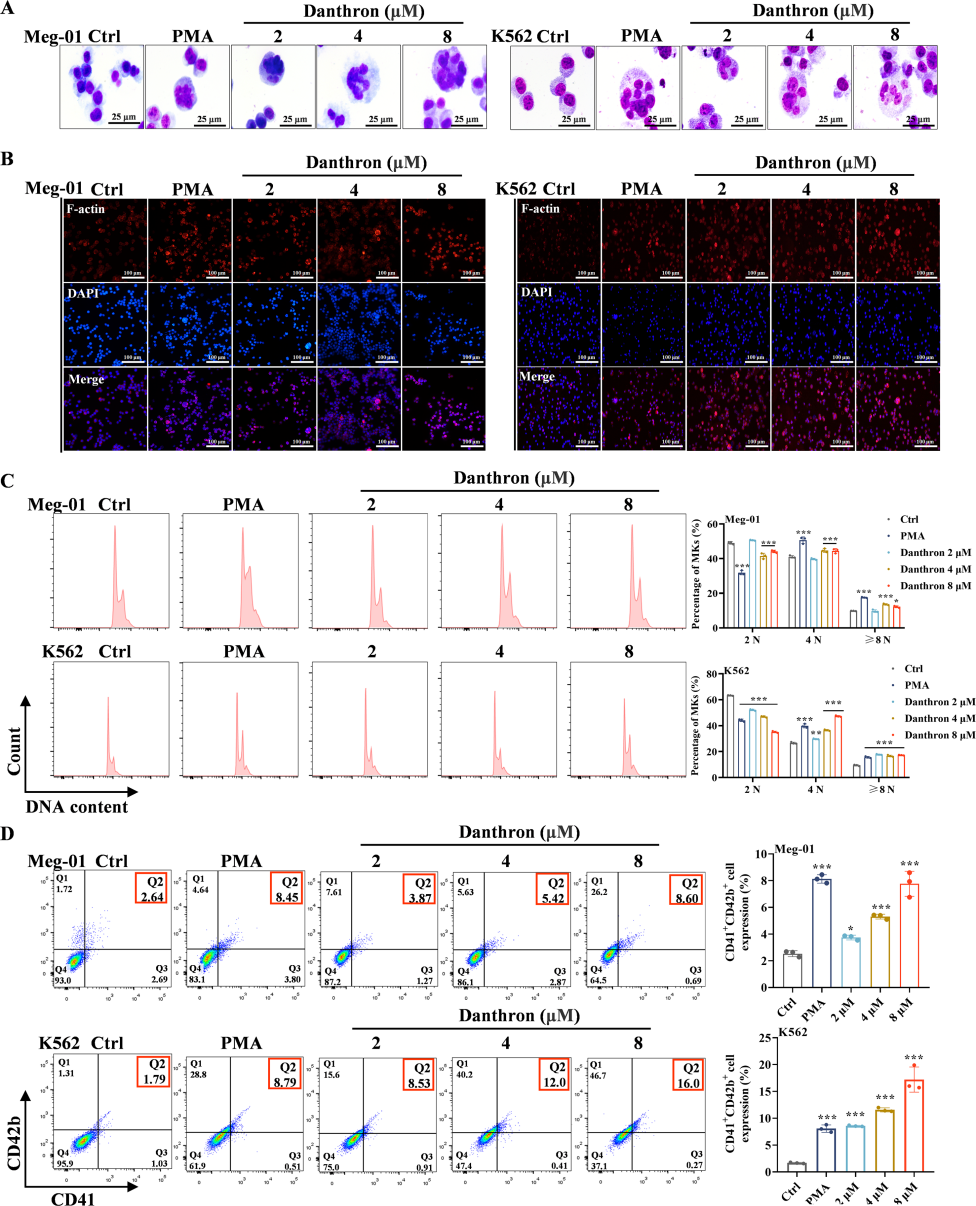
Danthron promotes MK differentiation and maturation *in vitro.* **(A)** Giemsa staining images of Meg-01 and K562 cells treated with danthron (2, 4 and 8 μM) and PMA (1.25 nM) on day 5. Scar bar: 25 µm. **(B)** Phalloidin staining reveals the expression of F-actin and multinuclear formation in each group after danthron (2, 4 and 8 μM) intervention on the 5th day of the two cells. Scar bar: 100 µm. **(C)** Analysis of DNA ploidy by flow cytometry. The histogram illustrates DNA ploidy. **(D)** The results of flow cytometry show the expression of CD41 and CD42b on the 5th day of the two cells with danthron (2, 4 and 8 μM) and PMA (1.25 nM) compared with the control group. All data are expressed as mean ± SD. n = 3 per group. *P < 0.05, **P < 0.01, and ***P < 0.001, vs. the Ctrl group.

To further elucidate the potential of danthron in promoting MK differentiation and maturation *in vitro*, we conducted a series of comprehensive experiments. MK maturation is characterized by distinctive features, including increased cell size, the formation of nuclear polyploidy, the development of demarcation membrane systems and cytoskeletal reorganization (26). Following a 5-day treatment of Meg-01 and K562 cells with danthron, we observed significant enhancements in cell size, nuclear polyploidy, and elevated expression levels of filamentous actin (F-actin) in MKs (**Figures 2A, B**). Furthermore, flow cytometry analysis of ploidy demonstrated a significant increase in the proportion of cells with 4N and ≥ 8N DNA content compared to the control group in both cell lines (**Figure 2C**). CD41 serves as a specific surface antigen of MKs, while CD42b is recognized as a marker of MK maturation (27). Treatment with danthron and PMA for 5 days resulted in a marked enhancement of the expression levels of both markers (**Figure 2D**). Collectively, these findings provide compelling evidence that danthron effectively promotes MK differentiation and maturation *in vitro*.

### 3.2 Danthron enhances the expression of transcription factors related to MK differentiation

**Figure 3:**
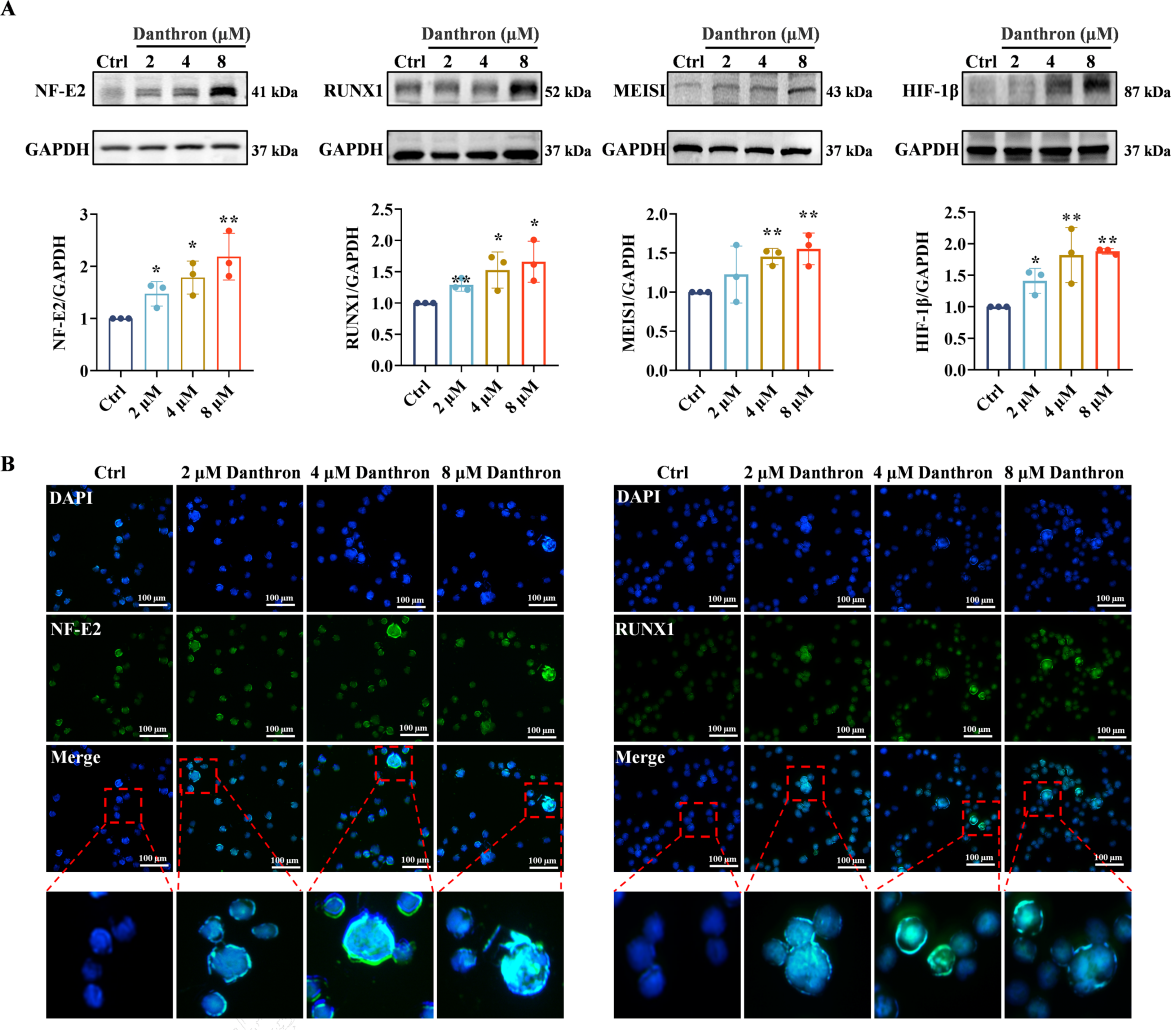
Danthron enhances the expression of transcription factors critical for regulating MK differentiation. **(A)** Western blot analysis of the expression of transcription factors related to the regulation of MK differentiation after 5 days of danthron treatment of Meg-01 cells. **(B)** Immunofluorescence images of transcription factors NF-E2 and RUNX1 on day 5 of danthron intervention in Meg-01 cells. Scar bar: 100 µm. All data are expressed as mean ± SD. n = 3 per group. *P < 0.05, **P < 0.01, vs Ctrl.

The differentiation and maturation of MK is a complex process that involves the precise regulation of various transcription factors, including RUNX1 (28), NF-E2 (29), MEIS1 (30) and HIF-1β (31). To investigate this, we assessed the expression levels of these transcription factors by western blot 5 days after danthron treatment in Meg-01 cells. The results revealed that danthron significantly enhanced the expression of these transcription factors (**Figure 3A**). Notably, RUNX1 and NF-E2 are essential for MK terminal maturation, facilitating critical processes such as polyploidization and cytoskeletal rearrangement, and they function as key transcriptional regulators of platelet release (9, 32). Immunofluorescence analysis further corroborated the upregulation of these transcription factors following danthron treatment (**Figure 3B**). Collectively, these findings provide robust evidence that danthron promotes the expression of transcription factors related to MK differentiation *in vitro*.

### 3.3 Danthron accelerates the recovery of platelet counts in mice with X-ray-induced thrombocytopenia

**Figure 4:**
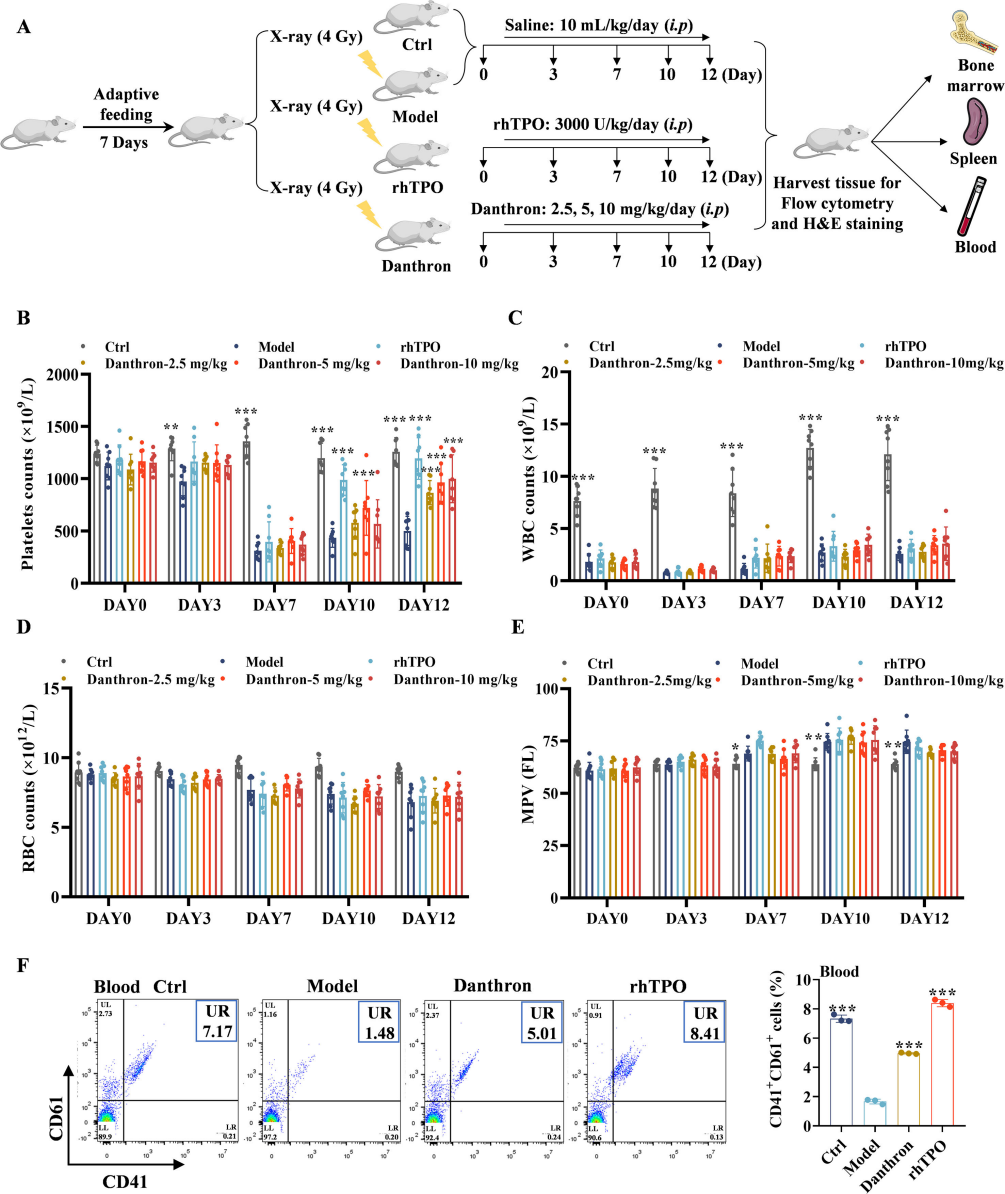
Danthron restores platelet counts in mice with thrombocytopenia. **(A)** Schematic representation of mouse groups, doses of radiation and administered. **(B-E)** Blood data showing **(B)** platelet, **(C)** WBC, **(D)** RBC and **(E)** MPV counts in each group of mice on days 1, 3, 7, 10 and 12 post-IR. All data are expressed as mean ± SD. n = 8 per group. *P < 0.05, **P < 0.01, ***P < 0.001, vs model. **(F)** Flow cytometric analysis of CD41 and CD61 expression in peripheral blood cells of mice on day 12. danthron: 10 mg/kg. All data are expressed as mean ± SD. n = 3 per group. *P < 0.05, **P < 0.01, ***P < 0.001, vs model. WBC: white blood cell, RBC, red blood cell; MPV, mean platelet volume.

Previously, we investigated the effects of danthron on MK differentiation and maturation *in vitro*. Building on those findings, we examined the role of danthron in promoting platelet production *in vivo* using a mouse model of RIT. The mice were divided into six groups: normal, model, rhTPO (3000 U/kg/day), and three danthron administration groups (2.5, 5, and 10 mg/kg/day). After a 7-day acclimatization period, all groups except the normal group were subjected to a single 4.0 Gy whole-body radiation exposure. The control and model groups received saline (10 mL/kg/day), while the experimental groups were treated with danthron and rhTPO for 12 consecutive days. Blood samples were collected from the ocular venous plexus on days 0, 3, 7, 10, and 12 (**Figure 4A**). Peripheral blood analysis revealed that white blood cell (WBC) counts significantly decreased in all groups except the control group on day 0 post-irradiation, and platelet counts reached their lowest levels on day 7, confirming the successful establishment of the RIT mouse model (**Figures 4B, C**). Platelet levels began to recover gradually in the experimental groups after the 7th day, with a more rapid recovery observed in the danthron and rhTPO-treated groups. By the 12th day of treatment, platelet counts in the danthron-treated group significantly exceed those in the model group (**Figure 4B**). Furthermore, there were no significant differences in WBCs, red blood cells (RBCs), or mean platelet volume (MPV) among the experimental groups (**Figures 4C, E**). Additionally, body weight and organ index of danthron-treated mice showed no significant deviations compared to other groups (**Supplementary Figures 1A–G**), indicating that danthron at these concentrations did not adversely affect other cell types or cause severe organ toxicity.

To further confirm the accelerated platelet recovery in the danthron group compared to model mice, we selected 10 mg/kg as the optimal danthron concentration for subsequent experiments. After 12 days of treatment, we observed a noticeable increase in peripheral blood platelets (CD41 and CD61) in mice treated with danthron (**Figure 4F**). Ultimately, these results demonstrate that danthron effectively restores platelet levels in mice suffering from thrombocytopenia.

### 3.4 Danthron enhances MK production in the bone marrow and spleen of mice with X-ray-induced thrombocytopenia

**Figure 5:**
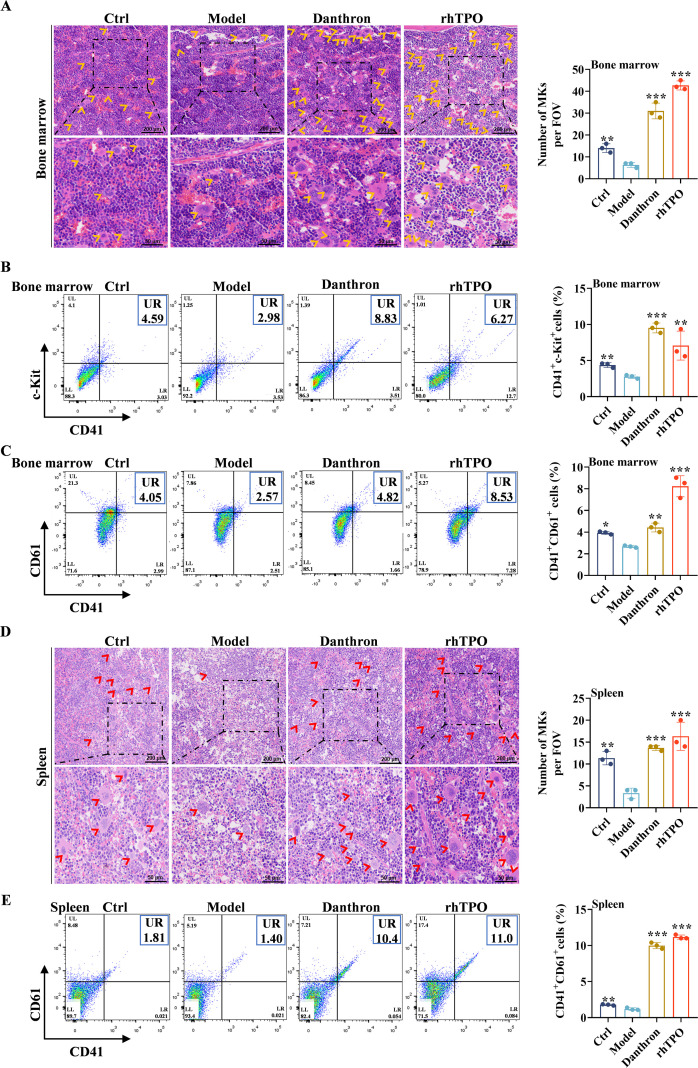
Danthron enhances MK counts in X-Ray-damaged bone marrow and spleen. **(A, D)** Representative images of bone marrow and spleen H&E staining with microscopic magnification of 200 × (top) and 100 × (bottom). The MKs are indicated by arrows. The histograms show the number of MKs in 3 random fields of view for each group. **(B, C, E)** Expression of c-Kit, CD41 and CD61 in bone marrow and CD41 and CD61 in spleen on day 12 of treatment by flow cytometry. All data are expressed as mean ± SD. n = 3 per group. *P < 0.05, **P < 0.01, ***P < 0.001, vs model. Danthron: 10 mg/kg.

Given that the bone marrow is the primary site of platelet production by MKs in the body (33), we employed flow cytometry to analyze surface markers associated with the MK lineage. Our findings indicated a significant increase in the number of MK progenitor cells (c-Kit^+^CD41^+^) and mature MKs (CD41^+^CD61^+^) in the bone marrow following treatment with danthron and rhTPO, compared to the model group (**Figures 5B, C**). Additionally, the spleen serves as a crucial organ for the clearance of aged or damaged platelets and contributes to platelet biogenesis (34). The co-expression of CD41^+^ and CD61^+^ in the spleen of danthron-treated mice was significantly elevated relative to the model group (**Figure 5E**). Furthermore, there were no evident pathological alterations observed in bone marrow and spleen tissues post-danthron treatment, accompanied by a notable increase in the number of MKs compared to the model group (**Figures 5A, D**). These findings suggest that danthron promotes platelet production both within and outside the bone marrow. Compared to the control group, dantron treatment did not lead to significant changes in serum ALT, AST, and CK levels in mice (**Supplementary Figures 4A–C**). In summary, the experimental data presented here demonstrate that danthron significantly enhances MK differentiation and platelet production in thrombocytopenic mice without inducing major toxicity or tissue damage.

### 3.5 Network pharmacology and molecular docking analyses uncover key targets and signaling pathways of danthron

**Figure 6:**
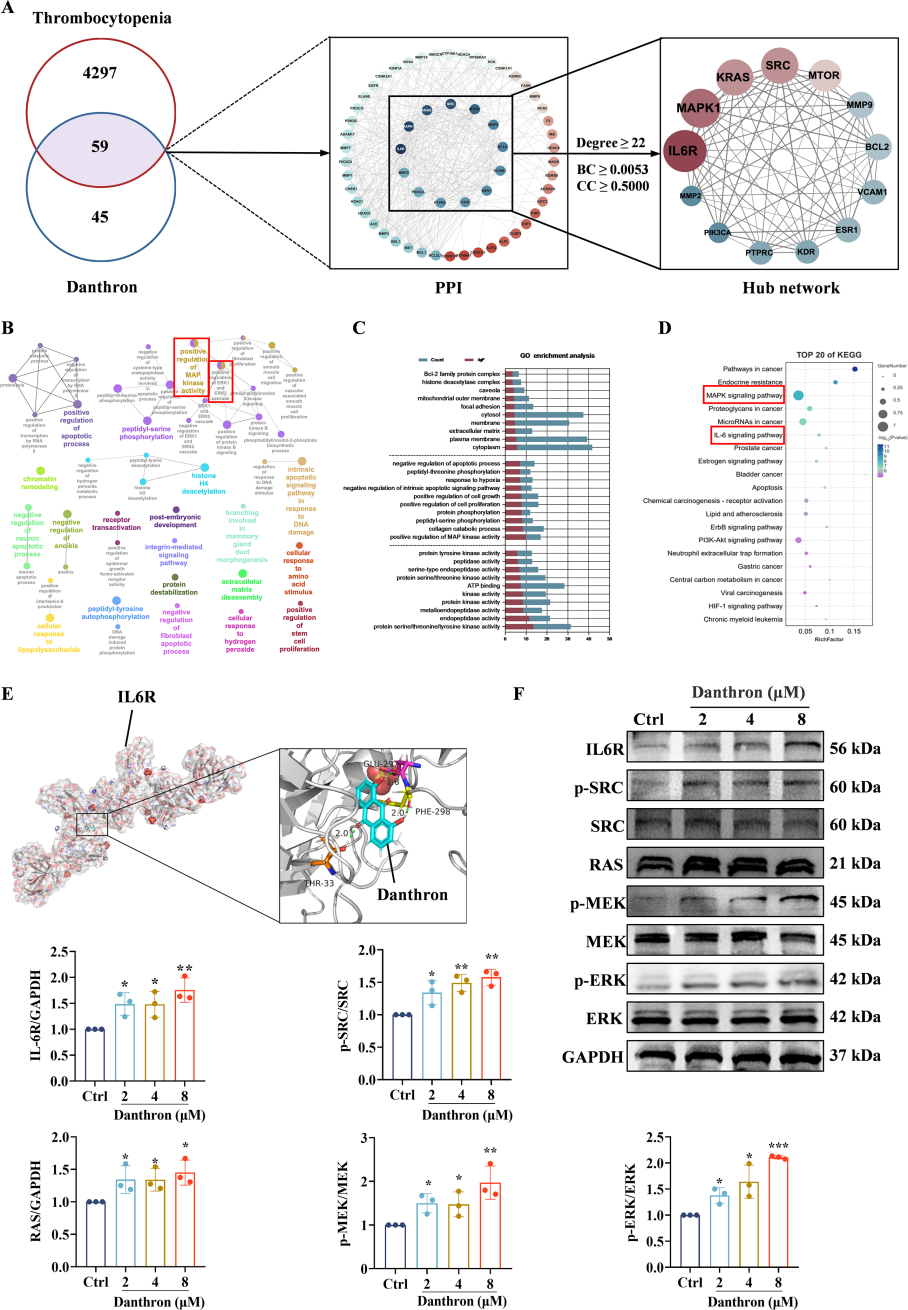
Targets and signaling pathway predicted for danthron treatment of thrombocytopenia using network pharmacology and molecular docking. **(A)** Common targets of danthron and thrombocytopenia Venn diagram (left), danthron-target-Thrombocytopenia network constructed by Cytoscape_v3.7.1 software and the protein-protein interaction (PPI) network of danthron with core targets of thrombocytopenia based on screening conditions of Degree ≥ 22, BC ≥ 0.0053, CC ≥ 0.5 (middle and right). **(B)** Visualization of cellular components, physiological processes and biofunctional enrichment analysis of potential therapeutic targets of danthron. **(C)** The top 10 MF terms with the most enriched danthron-related processes are arranged in ascending order of *P* value. **(D)** Top 20 molecular mechanisms of KEGG enrichment in thrombocytopenia treated with danthron. The pathway’s gene enrichment is represented by the size of the bubbles, while the range of *P* value is indicated by their color. **(E)** The molecular docking demonstrates the capability of danthron to bind to the core target IL-6R. **(F)** The detection of IL-6R, p-SRC, RAS, p-MEK and p-ERK1/2 in Meg-01 treated with no danthron and treated with danthron for 5 days by western blot. All data are expressed as mean ± SD. n = 3 per group. *P < 0.05, **P < 0.01, and ***P < 0.001, vs. the Ctrl group.

To investigate the potential targets of danthron for the treatment of thrombocytopenia, we conducted a comprehensive search of drug and disease target databases, identifying 4,356 disease targets and 104 drug targets. Among these, 59 common targets were considered as potential targets of danthron for the treatment of thrombocytopenia. Next, we used Cytoscape_v3.7.1 and set Degree ≥ 22, Betweenness centrality (BC) ≥ 0.0053, Closeness centrality (CC) ≥ 0.5000 as the filtering conditions, which led us to identify 13 core proteins, including IL-6R, MAPK1, KRAS, SRC, MTOR, MMP9, BCL2, VCAM1, ESR1, KDR, PTPRC, PIK3CA, MMP2 (**Figure 6A**).

Subsequently, we used GO and KEGG enrichment analyses to elucidate the mechanisms by which danthron may exert its effects in treating thrombocytopenia. The analysis revealed a total of 68 relevant GO terms associated with danthron (**Figure 6B**). These core targets were predominantly enriched in endopeptidase activity, protein kinase activity, positive regulation of MAP kinase activity, ATP binding, protein phosphorylation, cytoplasm, extracellular matrix, membrane and cytoplasmic membrane, which are very important for MK and platelet development and function. We ranked the top 10 molecular function (MF) terms associated with danthron in descending order of statistical significance (**Figure 6C**). KEGG enrichment analysis highlighted key pathways, specifically the MAPK and IL-6 signaling pathways, both of which are crucial for MK differentiation and platelet production (**Figure 6D**). To further validate the binding potential of danthron to these core targets, we employed molecular docking to evaluate the binding affinity to the core target IL-6R. The docking results indicated a binding score of 4.4742 kcal/mol, suggesting a strong affinity of danthron for this target (**Figure 6E**). Notably, KRAS, a member of the RAS family, plays a significant role in MK development, while RAS regulates numerous biological processes via the MAPK signaling pathway, including MK maturation and platelet production (35). Furthermore, the SRC tyrosine kinase family activates various downstream pathways, including the RAS/MAPK pathway, which are critical for cell proliferation, differentiation, and survival (36). Therefore, we hypothesized that danthron promotes MK differentiation and platelet production through the IL-6R/SRC/RAS/MAPK signaling axis. To test this hypothesis, we treated Meg-01 cells with danthron for 5 days *in vitro* and assessed the protein expression levels of IL-6R, p-SRC, RAS, p-MEK, and p-ERK via western blot analysis. The results demonstrated that danthron significantly enhanced the expression of these proteins compared to the control group (**Figure 6F**). In summary, these findings suggest that danthron may promote MK differentiation and treat thrombocytopenia by upregulating the expression of IL-6R, p-SRC, RAS, p-MEK, and p-ERK.

### 3.6 Danthron promotes MK differentiation through activation of IL-6R/SRC/RAS/MAPK pathway

**Figure 7:**
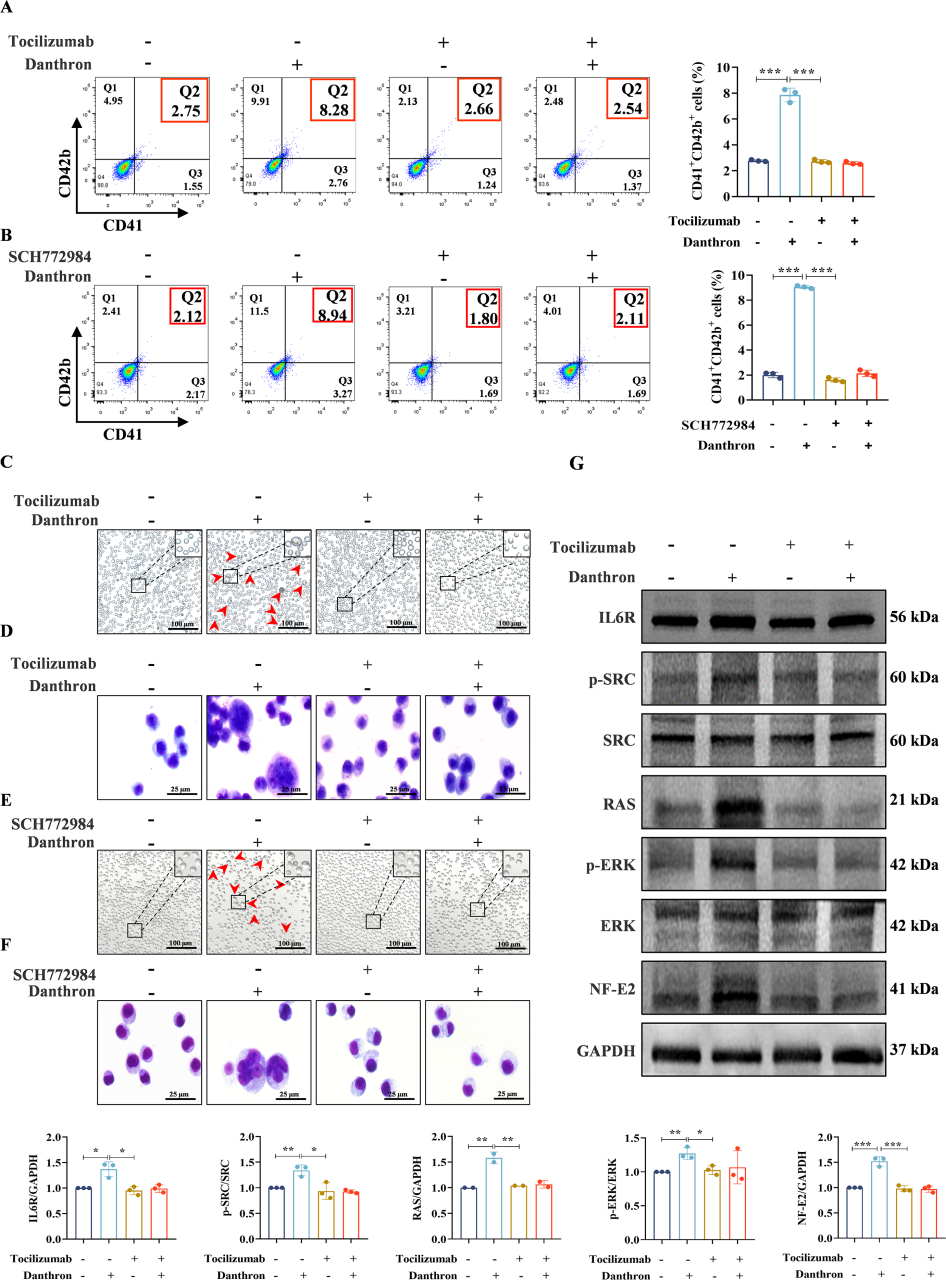
Danthron promotes MK differentiation and thrombopoiesis via IL-6R/SRC/RAS/MAPK signaling. **(A, B)** The flow cytometry analysis the expression of CD41 and CD42b on the 5th day of Meg-01 cells. The histogram illustrates the percentage of CD41^+^/CD42b^+^ cells. All data are expressed as mean ± SD. n = 3 per group. *P < 0.05, **P < 0.01, ***P < 0.001, vs danthron. **(C, E)** The representative images of Meg-01 of each group with different treatments on the 5th day. Scar bar: 100 µm, n = 3 per group. **(D, F)** Giemsa staining images of Meg-01 with different treatments on the 5th day. Scar bar: 25 µm, n = 3 per group. **(G)** Related pathway proteins expression of each group. All data are expressed as mean ± SD. n = 3 per group. *P < 0.05, **P < 0.01, ***P < 0.001, vs danthron.

To further elucidate the specific molecular mechanisms by which danthron regulates MK differentiation, we co-administered the IL-6R inhibitor (Tocilizumab, 100 ng/mL) and the ERK inhibitor (SCH772984, 1 μM) alongside danthron (8 μM) in Meg-01 cells for 5 days *in vitro*. Our results demonstrated that both Tocilizumab and SCH772984 significantly reduced the population of CD41^+^/CD42b^+^ cells (**Figures 7A, B**), as well as the formation of MK-like big cells and polyploidy (**Figures 7C–F**). Furthermore, to clarify the upstream-downstream relationship between IL-6R and the SRC/RAS/MAPK pathway, we employed Western blot analysis to assess the expression levels of p-SRC, RAS, p-MEK and p-ERK in Meg-01 cells following 5 days of co-intervention with Tocilizumab and danthron. The results indicated a decrease in the expression of all these proteins, thereby reinforcing the regulatory relationship between IL-6R and the SRC/RAS/MAPK pathway (**Figure 7G**). In addition, the expression of NF-E2, a critical transcription factor in MK differentiation, was significantly diminished (**Figure 7G**).

**Figure 8:**
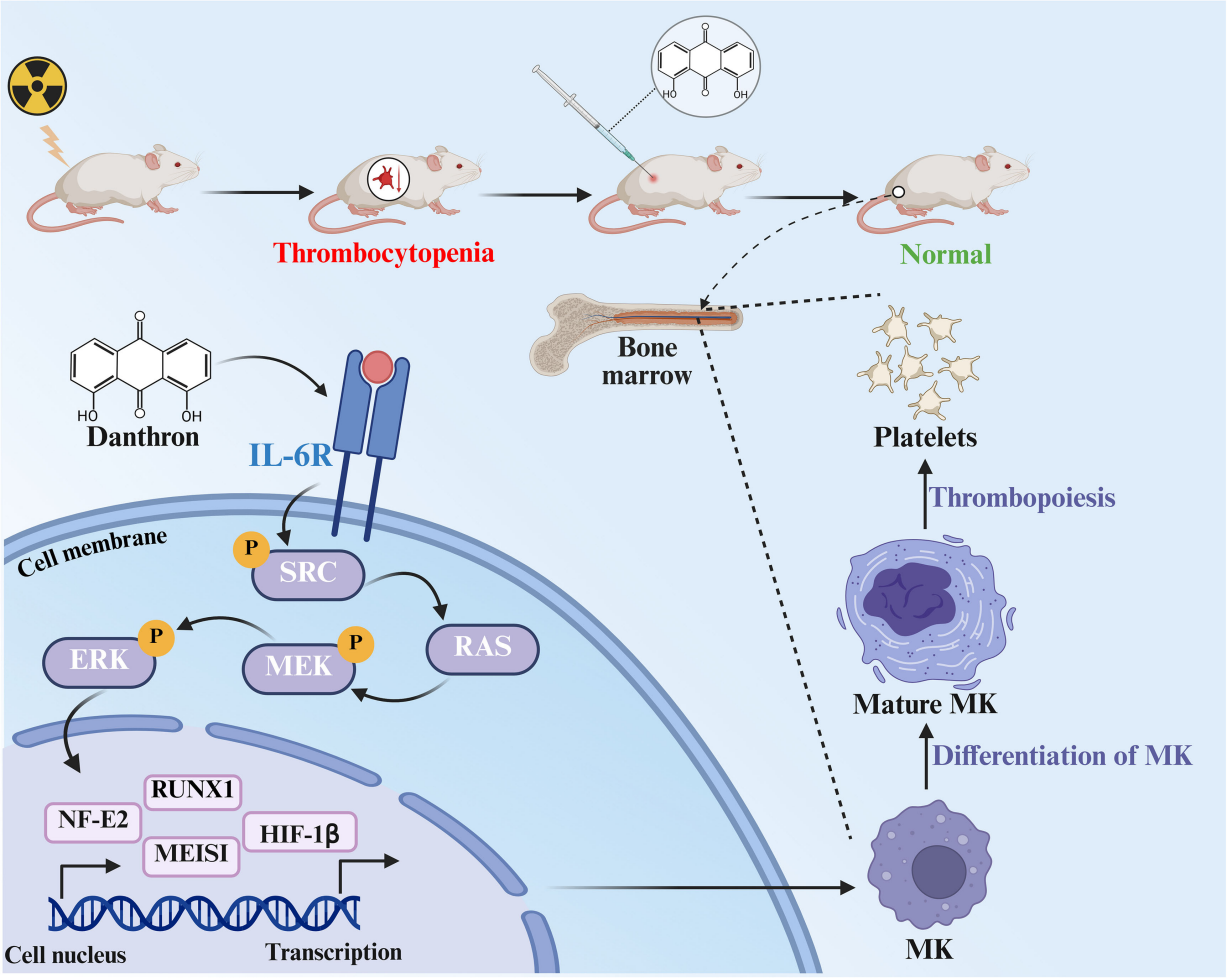
Schematic diagram of the mechanism by which Danthron regulates MK differentiation and platelet production.

In conclusion, danthron exerts its regulatory effect on MK differentiation and maturation by binding to IL-6R and subsequently activating the downstream SRC/RAS/MAPK signaling pathway.

## 4 Discussion

Thrombocytopenia is a prevalent complication in oncology and hematology, often limiting treatment intensity and worsening prognosis. Current agents, such as TPO-RAs and glucocorticoids, are associated with limited efficacy, cost constraints, or adverse events, underscoring the need for safer, mechanism-based alternatives. Based on previous discussions on the serious side effects of current clinical drugs and the traditional efficacy of rhubarb endowed with “promoting blood circulation and removing blood stasis” and “cooling blood and stopping bleeding”, we first identified that danthron, a representative anthraquinone derivative of rhubarb, is a novel natural small molecule IL-6R agonist that can promote MK differentiation and platelet production. Our findings demonstrated that danthron significantly enhanced MK maturation and upregulated the expression of key transcription factors associated with MK differentiation—NF-E2, MEIS1, RUNX1, and HIF-1β—without inducing significant cytotoxicity *in vitro.* Subsequently, we administered danthron intraperitoneally to mice with X-ray-induced thrombocytopenia for 12 days, during which we observed that danthron effectively promoted MK generation and restored platelet levels while exhibiting negligible toxicity to various organs. These results suggest that danthron possesses a pharmacological capability to regulate MK differentiation and platelet production safely, highlighting its potential as a therapeutic agent for thrombocytopenia.

The role of IL-6R and its downstream signaling pathways in the maturation and differentiation of MKs has been well established. Therefore, investigating IL-6R agonists or their natural regulators, such as monomers derived from traditional Chinese medicine, may provide novel therapeutic approaches for thrombocytopenia. Unlike TPO-RAs, danthron targets the IL-6R/SRC/RAS/MAPK axis, a signaling route with established roles in hematopoiesis but no clinically available agonists. In this study, we demonstrated that the binding of danthron to its target IL-6R not only induced SRC phosphorylation in a concentration-dependent manner but also activated the downstream RAS/MAPK signaling cascade. Therefore, we hypothesized that danthron regulates hematopoiesis through the IL-6R/SRC/RAS/MAPK pathway. To validate this hypothesis, we co-treated Meg-01 cells with IL-6R or ERK inhibitors alongside danthron. The results indicated that these inhibitors significantly diminished the effects of danthron, including the expression of downstream pathway proteins, the generation of MK-like large cells, polyploidy formation, and the proportion of CD41^+^/CD42b^+^ cells. These findings provide further support for our hypothesis.

Its mechanism is complementary to TPO-based therapies, raising the possibility of combination regimens. Although anthraquinones have been linked to dose-dependent toxicity, our short-term findings support a favorable safety window for danthron in hematopoietic applications. It should be noted that the reduced LDH release observed in Meg-01 cells on day 5 of danthron treatment does not necessarily indicate decreased cytotoxicity, as it may also result from changes in cell number, proliferation status, or cellular metabolic activity. The conclusions regarding the safety and cytotoxic effects of danthron in this study are therefore based on an integrated analysis of multiple lines of evidence, including additional functional assays and *in vivo* safety evaluations, rather than on LDH measurements alone. However, detailed toxicokinetic and chronic safety evaluations are warranted. Bridging basic medical research with clinical applications is of paramount importance, particularly regarding the complex process of determining initial human doses and their cross-species translation. The study by Nair et al. provides reliable data for clinical dose translation of danthron by converting animal doses to human-equivalent doses (37). Although danthron has not yet been employed in human clinical trials, its potential to induce MK differentiation and promote platelet production opens a promising new avenue for future thrombocytopenia treatments.

Looking ahead, we plan to pursue additional validation of the molecular mechanisms in murine models to solidify our conclusions. Furthermore, upon activation of the IL-6 pathway, the intracellular domain of IL-6R interacts with gp130, leading to receptor dimerization and the propagation of signaling through the MAPK pathway (38). However, the specific role of gp130 in the hematopoietic effects of danthron remains unclear. In the future, further exploration of the specific functions of IL-6R in MK differentiation and platelet production, as well as the regulatory mechanisms of IL-6R-related downstream signaling pathways in thrombopoiesis, will be of great importance. Additionally, while our murine RIT model closely mimics clinical thrombocytopenia, validation in human-derived CD34^+^ hematopoietic stem cells is essential to confirm translatability. It should be noted that the reduced proliferative activity observed in Meg-01 cells on day 5 following danthron treatment was interpreted in the context of other differentiation-related phenotypes. However, a decrease in proliferation alone does not establish a causal relationship with MK differentiation. Such changes may also reflect alterations in cell cycle dynamics or cellular metabolic states. In future studies, additional experiments, including live cell counting, cell cycle analysis, or the assessment of cell cycle arrest and differentiation markers, will be required to definitively establish causality. Nevertheless, in the present study, our interpretation is supported by multiple complementary lines of evidence, including enhanced expression of MK-specific markers, increased polyploidization, and consistent *in vivo* findings.

In conclusion, our findings not only validate the ethnopharmacological relevance of rhubarb but also identify danthron as a promising lead compound targeting IL-6R for thrombocytopenia therapy, providing new insights into the rational drug development for this disorder.

## 5 Conclusion

This study systematically demonstrates the pharmacological effects of danthron on MK differentiation and platelet production both *in vitro* and *in vivo*. Danthron markedly promotes MK maturation, as evidenced by increased expression of MK-specific markers, enhanced polyploidization, and improved platelet recovery in a mouse model of thrombocytopenia. Mechanistically, these effects are associated with activation of the IL-6R/SRC/RAS/MAPK signaling pathway. Importantly, *in vitro* and *vivo* safety evaluation indicates that danthron does not induce significant alterations in serum hepatic or cardiac biomarkers, nor does it cause evident pathological changes in the bone marrow or spleen or affect body weight and organ indices at the effective dose used in this study. Taken together, these findings suggest that danthron may serve as a promising pharmacological candidate for modulating MK differentiation and platelet production and provide a foundation for future studies exploring IL-6R–targeted therapeutic strategies.

## Data Availability

The raw data supporting the conclusions of this article will be made available by the authors, without undue reservation.

## References

[1] Kuter DJ . Treatment of chemotherapy-induced thrombocytopenia in patients with non-hematologic Malignancies. Haematologica. (2022) 107:1243–63. doi: 10.3324/haematol.2021.279512. PMID: 35642485 PMC9152964

[2] DiCarlo AL Poncz M Cassatt DR Shah JR Czarniecki CW Maidment BW . Development and licensure of medical countermeasures for platelet regeneration after radiation exposure. Radiat Res. (2011) 176:134–7. doi: 10.1667/rr2610.1. PMID: 21545289 PMC8370577

[3] DiCarlo AL Kaminski JM Hatchett RJ Maidment BW . Role of thrombocytopenia in radiation-induced mortality and review of therapeutic approaches targeting platelet regeneration after radiation exposure. J Radiat Oncol. (2016) 5:19–32. doi: 10.1007/s13566-015-0201-z. PMID: 41841152

[4] Yamasaki S . Bisphosphonate use for glucocorticoid-induced osteoporosis in older patients with immune thrombocytopenia: a clinical perspective. Ann Hematol. (2023) 102:1645–56. doi: 10.1007/s00277-023-05266-7. PMID: 37171596 PMC10175903

[5] Hou X Yan Z Liu S Gao N Chen J Wang Y . Corticosteroids plus metformin versus corticosteroids as front-line treatment for patients with newly diagnosed ITP and pre-existing type 2 diabetes mellitus: a multicentre propensity score-matched study. Br J Haematol. (2024) 206:1018–9. doi: 10.1111/bjh.19940. PMID: 39696781

[6] Martínez-Carballeira D Bernardo Á Caro A Soto I Gutiérrez L . Treatment of immune thrombocytopenia: contextualization from a historical perspective. Hematol Rep. (2024) 16:390–412. doi: 10.3390/hematolrep16030039. PMID: 39051412 PMC11270329

[7] Noh J . Megakaryopoiesis and platelet biology: roles of transcription factors and emerging clinical implications. Int J Mol Sci. (2021) 22:9615. doi: 10.3390/ijms22179615. PMID: 34502524 PMC8431765

[8] Tsutsumi N Masoumi Z James SC Tucker JA Winkelmann H Grey W . Structure of the thrombopoietin-MPL receptor complex is a blueprint for biasing hematopoiesis. Cell. (2023) 186:e22.4189–203. doi: 10.1016/j.cell.2023.07.037. PMID: 37633268 PMC10528194

[9] Li L Ni R Li Z Ming Y Liu L Peng D . Insights into regulatory factors in megakaryocyte development and function: basic mechanisms and potential targets. Front Biosci (Landm Ed). (2022) 27:313. doi: 10.31083/j.fbl2711313. PMID: 36472109

[10] Nishimura S Nagasaki M Kunishima S Sawaguchi A Sakata A Sakaguchi H . IL-1α induces thrombopoiesis through megakaryocyte rupture in response to acute platelet needs. J Cell Biol. (2015) 209:453–66. doi: 10.1083/jcb.201410052. PMID: 25963822 PMC4427781

[11] Ng AP Kauppi M Metcalf D Hyland CD Josefsson EC Lebois M . Mpl expression on megakaryocytes and platelets is dispensable for thrombopoiesis but essential to prevent myeloproliferation. Proc Natl Acad Sci USA. (2014) 111:5884–9. doi: 10.1073/pnas.1404354111. PMID: 24711413 PMC4000844

[12] Hacein-Bey-Abina S Estienne M Bessoles S Echchakir H Pederzoli-Ribeil M Chiron A . Erythropoietin is a major regulator of thrombopoiesis in thrombopoietin-dependent and -independent contexts. Exp Hematol. (2020) 88:15–27. doi: 10.1016/j.exphem.2020.07.006. PMID: 32721504

[13] Kim H Lee M-K Kim HR . Difference in megakaryocyte expression of GATA-1, IL-6, and IL-8 associated with maintenance of platelet counts in patients with plasma cell neoplasm with dysmegakaryopoiesis. Exp Hematol. (2019) 73:e2.13–7. doi: 10.1016/j.exphem.2019.02.005. PMID: 30825517

[14] Webb CE Vautrinot J Hers I . IL-6 as a mediator of platelet hyper-responsiveness. Cells. (2025) 14:766. doi: 10.3390/cells14110766. PMID: 40497942 PMC12153796

[15] Wen Y Yan P Fan P Lu S Li M Fu X . The application of rhubarb concoctions in traditional chinese medicine and its compounds, processing methods, pharmacology, toxicology and clinical research. Front Pharmacol. (2024) 15:1442297. doi: 10.3389/fphar.2024.1442297. PMID: 39170703 PMC11335691

[16] Liudvytska O Ponczek MB Krzyżanowska-Kowalczyk J Kowalczyk M Balcerczyk A Kolodziejczyk-Czepas J . Effects of rheum rhaponticum and rheum rhabarbarum extracts on haemostatic activity of blood plasma components and endothelial cells *in vitro*. J Ethnopharmacol. (2023) 315:116562. doi: 10.1016/j.jep.2023.116562. PMID: 37201663

[17] Lin W Hou J Han T Zheng L Liang H Zhou X . Efficacy and safety of traditional chinese medicine for intracranial hemorrhage by promoting blood circulation and removing blood stasis: a systematic review and meta-analysis of randomized controlled trials. Front Pharmacol. (2022) 13:942657. doi: 10.3389/fphar.2022.942657. PMID: 36249750 PMC9553997

[18] Chen Y Zhang Y . Interpretation of the pathological mechanism of blood stasis in traditional chinese medicine in light of understanding of hypercoagulable states in modern medicine. Chin Med Natural Pro. (2025) 5:e30–e4. doi: 10.1055/s-0045-1806864. PMID: 41839210

[19] Shi X Yue S Tang Y Chen Y Zhou G Zhang J . A network pharmacology approach to investigate the blood enriching mechanism of Danggui buxue decoction. J Ethnopharmacol. (2019) 235:227–42. doi: 10.1016/j.jep.2019.01.027. PMID: 30703496

[20] Li X Chu S Liu Y Chen N . Neuroprotective effects of anthraquinones from rhubarb in central nervous system diseases. Evid Based Complem Alternat Med. (2019) 2019:3790728. doi: 10.1155/2019/3790728. PMID: 31223328 PMC6541978

[21] Shi X Zhang Y Lin B Zhou Y Suo W Wei J . Danthron attenuates experimental atherosclerosis by targeting foam cell formation. Exp Physiol. (2021) 106:653–62. doi: 10.1113/ep089021. PMID: 33450102

[22] Cao Y Pu Z Tang Y Shen J Chen Y Kang A . Advances in bio-active constituents, pharmacology and clinical applications of rhubarb. Chin Med. (2017) 12:36. doi: 10.1186/s13020-017-0158-5. PMID: 29299052 PMC5745730

[23] Redondo PC Granados MP Fernández E Collado-Pérez MA Teruel-Montoya R Ferrer-Marín F . EFCAB13 is a novel age-dependent promotor of SOCE in blood platelets and megakaryoblastic MEG-01 cells. Thromb Haemost. (2025). doi: 10.1055/a-2689-7785. PMID: 40972679

[24] Huang Y Su S Chuang H Chen H Twu Y . Histone deacetylation-regulated cell surface Siglec-7 expression promoted megakaryocytic maturation and enhanced platelet-like particle release. J Thromb Haemost. (2023) 21:329–43. doi: 10.1016/j.jtha.2022.11.007. PMID: 36700509

[25] Raghuwanshi S Dahariya S Sharma DS Kovuru N Sahu I Gutti RK . RUNX1 and TGF-β signaling cross talk regulates Ca^2+^ ion channels expression and activity during megakaryocyte development. FEBS J. (2020) 287:5411–38. doi: 10.1111/febs.15329. PMID: 32281291

[26] Geue S Aurbach K Manke MC Manukjan G Münzer P Stegner D . Pivotal role of PDK1 in megakaryocyte cytoskeletal dynamics and polarization during platelet biogenesis. Blood. (2019) 134:1847–58. doi: 10.1182/blood.2019000185. PMID: 31578203

[27] Ono M Matsubara Y Shibano T Ikeda Y Murata M . GSK-3β negatively regulates megakaryocyte differentiation and platelet production from primary human bone marrow cells *in vitro*. Platelets. (2011) 22:196–203. doi: 10.3109/09537104.2010.541959. PMID: 21231855

[28] Wang C Tu Z Cai X Wang W Davis AK Nattamai K . A critical role of RUNX1 in governing megakaryocyte-primed hematopoietic stem cell differentiation. Blood Adv. (2023) 7:2590–605. doi: 10.1182/bloodadvances.2022008591. PMID: 36661340 PMC10250926

[29] Avanzi MP Davila JG Goldberg F Mitchell WB . C-MYC and NF-E2 genes regulate proplatelet formation in cultured megakaryocytes with different levels of polyploidization. Blood. (2012) 120:2289. doi: 10.1182/blood.V120.21.2289.2289. PMID: 41761659

[30] Zeddies S Jansen SB Di Summa F Geerts D Zwaginga JJ Van Der Schoot CE . MEIS1 regulates early erythroid and megakaryocytic cell fate. Haematologica. (2014) 99:1555–64. doi: 10.3324/haematol.2014.106567. PMID: 25107888 PMC4181251

[31] Wang L Liu S Luo J Mo Q Ran M Zhang T . Targeting a thrombopoietin-independent strategy in the discovery of a novel inducer of megakaryocytopoiesis, DMAG, for the treatment of thrombocytopenia. Haematologica. (2023) 108:1394–411. doi: 10.3324/haematol.2022.282209. PMID: 36546424 PMC10153531

[32] Noetzli LJ French SL Machlus KR . New insights into the differentiation of megakaryocytes from hematopoietic progenitors. Arterioscler Thromb Vasc Biol. (2019) 39:1288–300. doi: 10.1161/atvbaha.119.312129. PMID: 31043076 PMC6594866

[33] Stone AP Nascimento TF BarraChina MN . The bone marrow niche from the inside out: how megakaryocytes are shaped by and shape hematopoiesis. Blood. (2022) 139:483–91. doi: 10.1182/blood.2021012827. PMID: 34587234 PMC8938937

[34] Valet C Magnen M Qiu L Cleary SJ Wang KM Ranucci S . Sepsis promotes splenic production of a protective platelet pool with high CD40 ligand expression. J Clin Invest. (2022) 132:e153920. doi: 10.1172/jci153920. PMID: 35192546 PMC8970674

[35] Pawinwongchai J Mekchay P Nilsri N Israsena N Rojnuckarin P . Regulation of platelet numbers and sizes by signaling pathways. Platelets. (2021) 32:1073–83. doi: 10.1080/09537104.2020.1841893. PMID: 33222582

[36] Raji L Tetteh A Amin A . Role of c-Src in carcinogenesis and drug resistance. Cancers (Basel). (2023) 16:32. doi: 10.3390/cancers16010032. PMID: 38201459 PMC10778207

[37] Nair AB Jacob S . A simple practice guide for dose conversion between animals and human. J Bas Clin Pharm. (2016) 7:27–31. doi: 10.4103/0976-0105.177703. PMID: 27057123 PMC4804402

[38] Uciechowski P Dempke WCM . Interleukin-6: a masterplayer in the cytokine network. Oncology. (2020) 98:131–7. doi: 10.1159/000505099. PMID: 31958792

