# Supplementary material for: Danthron as a novel IL-6R agonist promotes thrombopoiesis via the SRC/RAS/MAPK pathway
Source: Front Immunol. 2026 Mar 27;17:1730028. doi: 10.3389/fimmu.2026.1730028 (PMC13065674; doi:10.3389/fimmu.2026.1730028)
Supplement: Supplementary file 2 [file DataSheet2.pdf]

Supplementary Material

1 Supplementary Figures

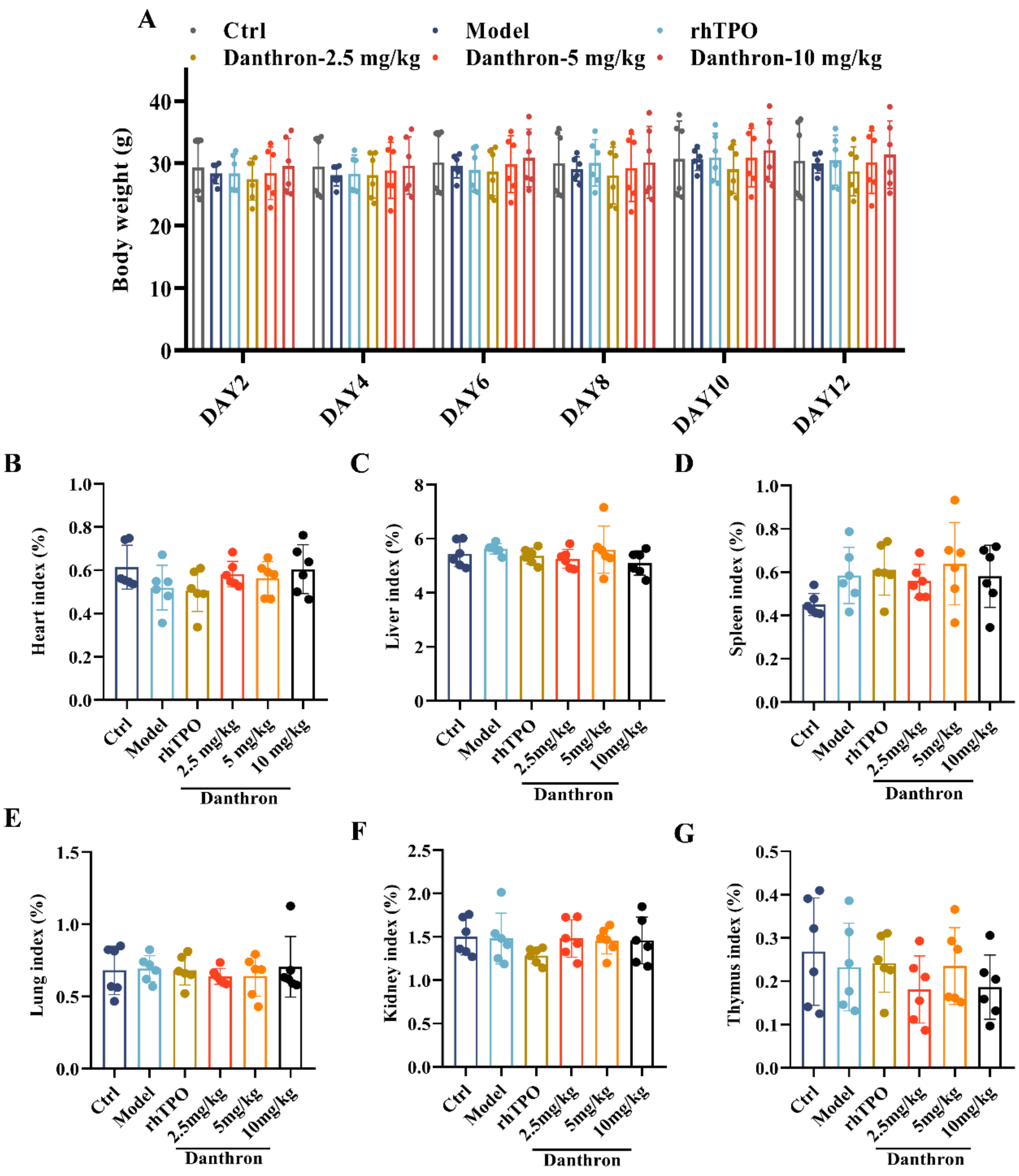

Supplementary Figure 1. Danthron treatment had no significant effect on body weight or organ indices in mice. **(A)** Body weights were measured and recorded on days 2, 4, 6, 8, 10, and 12 after administration (n = 6). **(B–G)** Organ weights were measured on day 12 of administration (n = 6). Data are presented as mean ± SD. Statistical analysis was performed using two-way ANOVA (for body

weight) and one-way ANOVA (for organ indices). No statistically significant differences were observed among groups (*P* > 0.05 vs. model group).

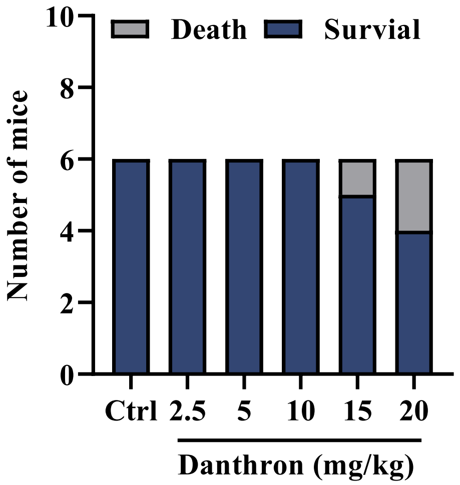

Supplementary Figure 2. Toxic effects of different doses of danthron on the survival of normal mice. Each group consisted of 6 mice (3 males and 3 females). Statistical analysis was performed using two-way ANOVA.

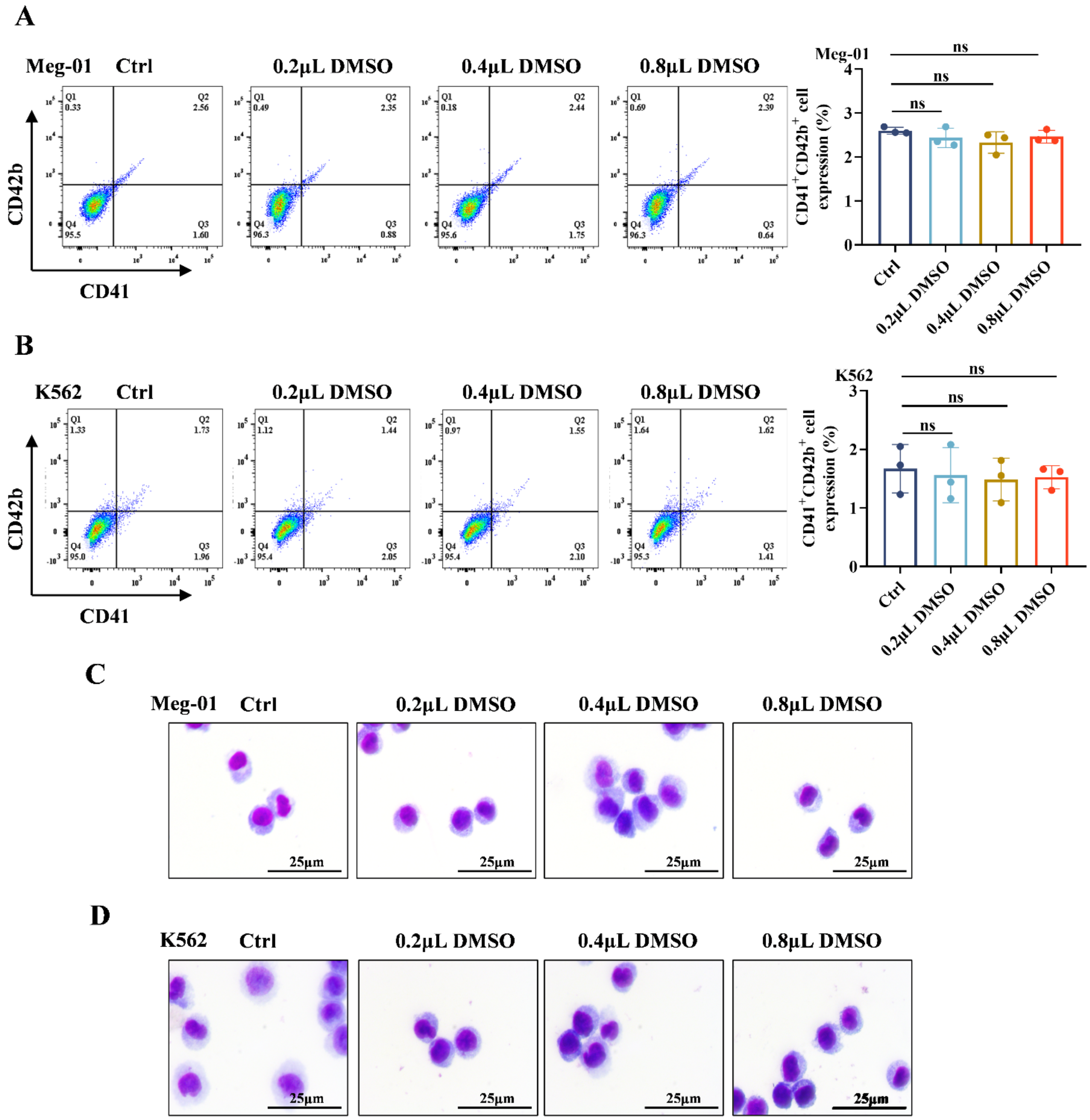

Supplementary Figure 3. Effects of DMSO on MK differentiation of Meg-01 and K562 cells *in vitro*. **(A, B)** The results of flow cytometry show the expression of CD41 and CD42b on the 5th day of Meg-01**(A)** and K562 **(B)** cells with DMSO (0.2, 0.4 and 0.8 μL). **(C, D)** Giemsa staining images of Meg-01 **(C)** and K562 **(D)** cells treated with DMSO (0.2, 0.4 and 0.8 μL) on day 5. Scar bar: 25 μm. All data are expressed as mean ± SD. n = 3 per group. No statistically significant differences were observed among groups ($P > 0.05$ vs. Ctrl group).

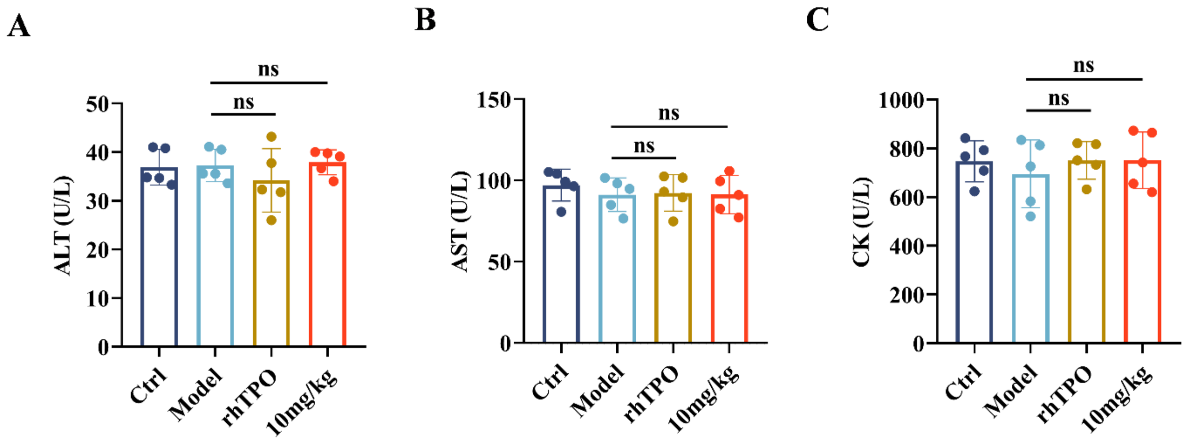

Supplementary Figure 4. Effects of danthron on serum ALT, AST, and CK levels in mice. The results show the levels of ALT **(A)**, AST **(B)** and CK **(C)** measured using a fully automated biochemical analyzer. Data are presented as mean ± SD (n = 5 per group). No significant differences were observed between the danthron -treated group and the model group (*P* > 0.05 vs. model group).

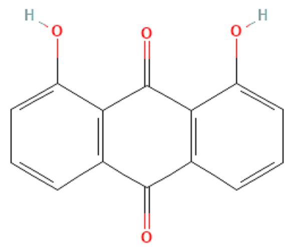

Supplementary Figure 5. Chemical structure of danthron. The chemical structure of danthron (1,8-dihydroxyanthraquinone), an anthraquinone derivative with the molecular formula $C_{14}H_8O_4$, was obtained from the PubChem database.

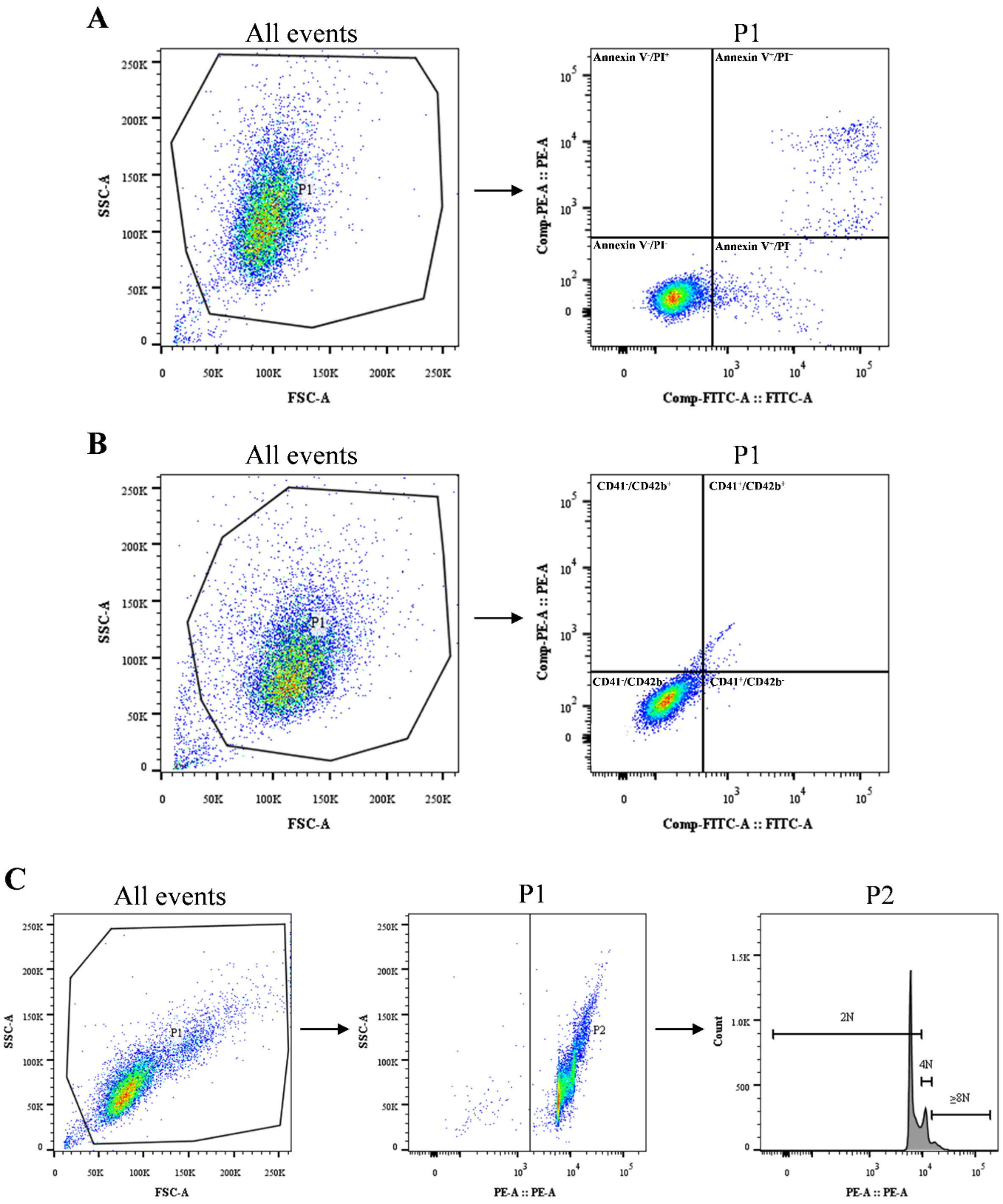

Supplementary Figure 6. Representative gating strategies in *in vitro* flow cytometry. **(A)** Gating strategy for apoptosis. **(B)** Gating strategy for CD41/CD61 expression in K562 cells. **(C)** Gating strategy for cell ploidy.

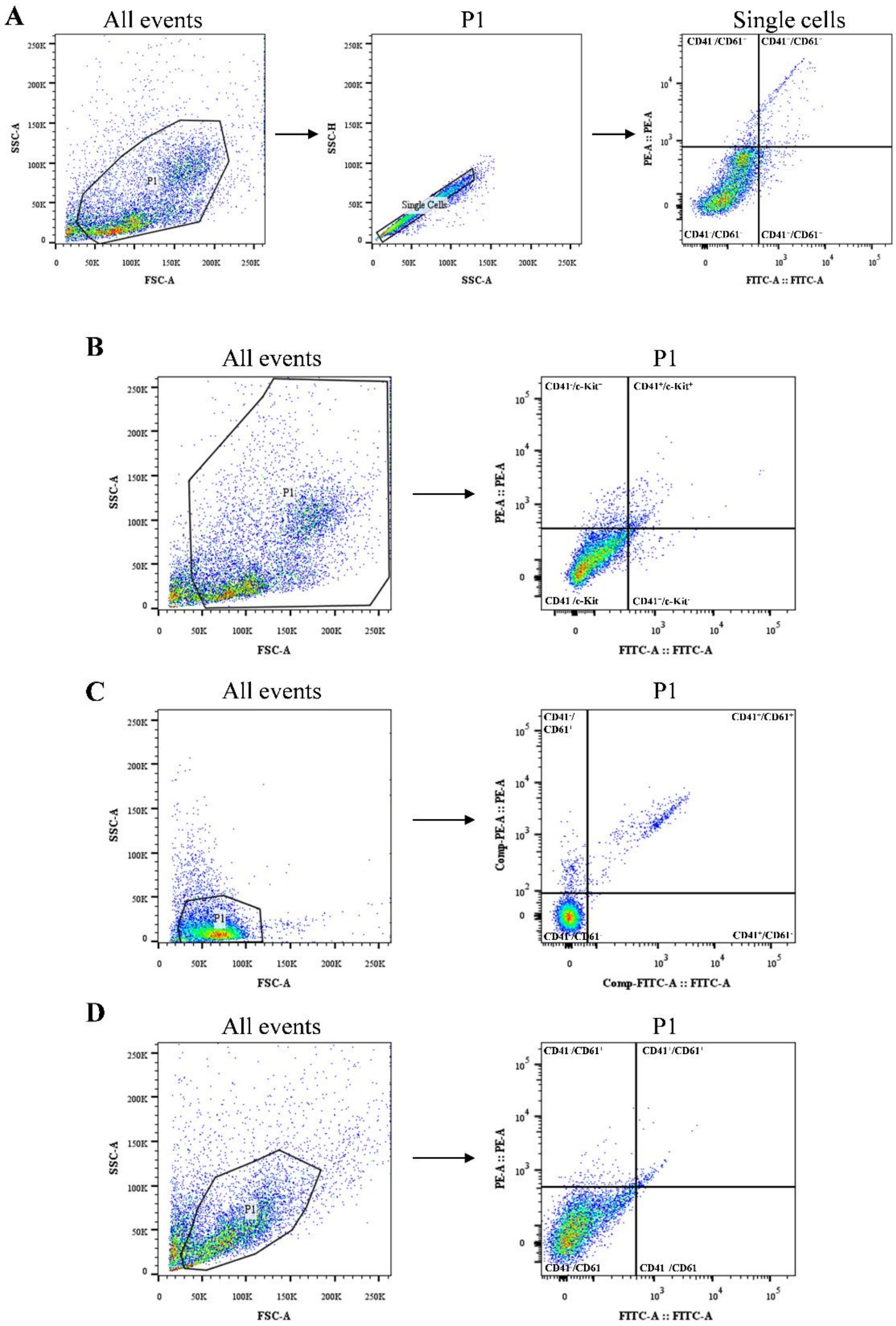

Supplementary Figure 7. Representative gating strategies in mouse *in vivo* flow cytometry. **(A)** Gating strategy for bone marrow megakaryocyte progenitor cells (CD41$^+$/c-Kit$^+$). **(B)** Gating strategy for bone marrow megakaryocytes (CD41$^+$/CD61$^+$). **(C)** Gating strategy for peripheral blood platelets (CD41$^+$/CD61$^+$). **(D)** Gating strategy for spleen megakaryocytes (CD41$^+$/CD61$^+$).
